# Tumor-associated macrophages in nanomaterial-based anti-tumor therapy: as target spots or delivery platforms

**DOI:** 10.3389/fbioe.2023.1248421

**Published:** 2023-08-16

**Authors:** Jixuan Zheng, Jinting Jiang, Yicheng Pu, Tingrui Xu, Jiantong Sun, Qiang Zhang, Ling He, Xiao Liang

**Affiliations:** ^1^ Key Laboratory of Birth Defects and Related Diseases of Women and Children, Ministry of Education, West China Second Hospital, West China School of Medicine, West China School of Pharmacy, Sichuan University, Chengdu, China; ^2^ Hospital of Chengdu University of Traditional Chinese Medicine, Chengdu, China

**Keywords:** tumor-associated macrophage, nanoparticle, tumor micorenvironment targeting, nanoparticle delivery, antitumor therapy

## Abstract

Targeting tumor-associated macrophages (TAMs) has emerged as a promising approach in cancer therapy. This article provides a comprehensive review of recent advancements in the field of nanomedicines targeting TAMs. According to the crucial role of TAMs in tumor progression, strategies to inhibit macrophage recruitment, suppress TAM survival, and transform TAM phenotypes are discussed as potential therapeutic avenues. To enhance the targeting capacity of nanomedicines, various approaches such as the use of ligands, immunoglobulins, and short peptides are explored. The utilization of live programmed macrophages, macrophage cell membrane-coated nanoparticles and macrophage-derived extracellular vesicles as drug delivery platforms is also highlighted, offering improved biocompatibility and prolonged circulation time. However, challenges remain in achieving precise targeting and controlled drug release. The heterogeneity of TAMs and the variability of surface markers pose hurdles in achieving specific recognition. Furthermore, the safety and clinical applicability of these nanomedicines requires further investigation. In conclusion, nanomedicines targeting TAMs hold great promise in cancer therapy, offering enhanced specificity and reduced side effects. Addressing the existing limitations and expanding our understanding of TAM biology will pave the way for the successful translation of these nano-therapies into clinical practice.

## 1 Introduction

The association between tumors and inflammation was first proposed in the 19th century, in terms of the fact that tumors often arose at the sites of chronic inflammation. Actually, it has been demonstrated that inflammation is closely associated with all stages of development and the malignant progression of tumors (Zhao et al., 2021). The tumor microenvironment (TME) is an ensemble of environments for the survival of tumor cells, which includes the surrounding extracellular matrix, blood vessels, malignant cells, endothelial cells, stromal cells, immune cells, and signaling molecules (Bader et al., 2020; Cheng N et al., 2021). Together with other pro-tumor factors in TME, inflammatory immune cells shape a pro-tumoral environment where sustained cell proliferation certainly potentiates and promotes the risk of tumor (Coussens and Werb, 2002).

Tumor-associated macrophages (TAMs) are the most abundant immune cells in the TME of many tumors (Santander et al., 2015; Wang H. et al., 2021; Xiang et al., 2021) and have been shown to promote tumor cell proliferation, induce immunosuppression, promote tumor metastasis, invasion, and intravasation, thereby promoting tumor development and malignancy (Vitale et al., 2019; Cassetta and Pollard, 2020). At the site of tumor pathogenesis, infiltration of macrophages was found to be associated with chemoresistance as well as poor prognosis in tumors. At the metastatic site, macrophages are able to promote the extravasation, survival, and growth of tumor cells (Qian and Pollard, 2010).

TAMs are mainly derived from monocytes in the blood and belong to the monocyte-macrophage system. Mononuclear macrophages have a significant impact on TAMs’ accumulation, which serve as an important component of the monocyte-macrophage system. Studies have found that the recruitment of circulating monocytes helps promote the local accumulation of TAMs (Franklin et al., 2014). Multiple chemokines (such as C-C motif chemokine 2(CCL2)) and cytokines (such as members of the vascular endothelial growth factor family) can recruit circulating inflammatory monocytes into tumor tissue. The growth of tumor tissue can also induce CC chemokine receptor 2 positive (CCR2+) monocytes to differentiate into TAMs (Pan et al., 2020).

In response to local cues, macrophages are usually activated into two different phenotypes: “M1” phenotypes and “M2” phenotypes (Boutilier and Elsawa, 2021). M1 macrophages, named classically activated macrophages, were proinflammatory and reported to play central roles in anti-tumor immunity. Usually, M1 macrophages are characterized by induction of inducible nitric oxide synthase (iNOS) and pro-inflammatory cytokines (e.g., tumor necrosis factor-α(TNF-α), interleukin-1β(IL-1β), IL-6, and IL-12) and enhanced phagocytic ability. Actually, in response to TME cues, most of the TAMs are activated into an M2 (alternatively activated) phenotype, which is anti-inflammatory and reported to suppress anti-tumor immunity and promote tumor growth and metastasis (Cassetta and Pollard, 2020; Dancsok et al., 2020). These M2 macrophages are characterized by the production of immunosuppressive cytokines (e.g., IL-10, IL-1ra, and transforming growth factor (TGF-β)), and upregulation of cell surface molecules (e.g., CD206). M2-type TAMs are capable of interacting with tumor cells via exosomes or secreting a variety of cytokines. In this way, they can recruit regulatory T (Treg) cells to interrupt immune cell interactions to suppress the antitumor immune response of T cells and create an immunosuppressive microenvironment, thereby promoting tumor cell proliferation, invasion, migration, and angiogenesis (Takenaka et al., 2019). In addition, TAMs also interact with the microbiota in colorectal cancer (CRC) through multiple metabolic pathways to function as immunosuppressive agents, thus promoting the escape of tumor cells into the blood and inhibiting antitumor immune responses (Wang H. et al., 2021; Jiang et al., 2022). Recent studies have also identified that TAMs may potentiate, mediate, or antagonize the antitumor activity of radiation, cytotoxic agents, and checkpoint inhibitors (Chen D. et al., 2021).

Studies have proved that TAMs are important for tumor prognosis and metastasis, drug resistance and metabolism, as well as changes in the TME (Cheng N et al., 2021). Therefore, TAM is an important factor that deserves attention in tumor therapy. The fluidity and distribution specificity of TAMs enables them to specifically transfer drugs, enhance local drug accumulation, and deepen drug penetration. In addition, the secretory ability of TAMs also contributes to amplifying the effects of drugs (Pan et al., 2020).

Due to the close association between tumor cells and TAMs, TAMs may be an ideal target for modulating the TME and tumor cells. In our study, we conducted a systematic review of antitumor nanomedicines targeting TAMs from aspects including inhibiting the recruitment of TAMs, inhibiting the survival of TAMs, promoting the switch of TAM phenotypes, and targeting molecular techniques. In addition, we reviewed macrophage-based drug delivery systems. Systematically, we summarized the latest progression in TAM-based antitumor nanotechnology, providing promising ideas to modify or even design effective drug carriers.

## 2 Targeting TAMs: a promising spot owing to their vital roles in TME

In the past few decades, immune therapy has come to the fore in the treatment of several malignancies as an effective and promising antitumor method (Sadeghi Rad et al., 2021). The clinical response toward immunotherapeutic intervention was observed in a variety of tumors (Sadeghi Rad et al., 2021; Xu et al., 2022). However, more patients receive little clinical benefit and are awaiting improved solutions (Binnewies et al., 2018). To develop new antitumor strategies, researchers have paid much attention to the TME, where tumor cells reside, due to its complex and diverse immune components. As one of the most crucial parts of the TME, TAMs promote tumor progression in several ways.

1) TAMs participate in tumor angiogenesis. TAMs have been identified as a key inducer of angiogenesis, which assures the supply of nutrition and oxygen to the tumor sites. Chiefly, TAM-derived cytokines like vascular endothelial growth factors (VEGF), TNFα and IL1α promote angiogenesis by enhancement of endothelial cell (EC) proliferation, chemotaxis, etc., [29, 30]. Besides, it has been demonstrated that TAM-derived matrix metalloproteinases (MMPs) and total proteins (TPs) also increase intratumoral microvessel density (Nakamura et al., 2007; Kawahara, 2010). Additionally, chitinase-3-like-1 (YKL-40) enhances MAPK signaling in ECs, resulting in upregulating vascular endothelial growth factor receptor-1 (VEGFR-1) and vascular endothelial growth factor receptor-2 (VEGFR-2) and thus promoting vessel sprouting (Shao, 2013). Tie2-expressing TAMs have been thought significant in tumor angiogenesis but recent studies are challenging that (Zhang and Brekken, 2022).
2) TAMs modulate tumor immunoregulation. TAMs perform immunosuppression capability mainly by inhibiting activation and proliferation of CD8^+^ T cells by releasing cytokines like prostaglandin E2(PGE2), TGF-β, IL-10, CCL20, and arginase 1 (ARG1)[32–35], directly hindering the viability of immune cells or indirectly inhibiting the recruitment of them [36].
3) TAMs enhance tumor resistance. TAMs reduce the influence of chemotherapy drugs mainly through the secretion of enzymes and cytokines. Researchers have reported TAM-derived cathepsins preventing Taxol-induced tumor cell apoptosis and cytokines IL-6 and IL-10 weakening the chemotherapy effect of doxorubicin [37, 38]. In addition, the TAM-induced abnormal blood vessels hinder drug delivery toward tumor tissue (Liu J. et al., 2023).
4) TAMs promote Cancer Stem Cells (CSCs). A study shows that CSCs play a specific role in inducing the expression of milk-fat globule-epidermal growth factor-VIII (MFG-E8) in TAMs. In return, these TAM-derived factors promote tumorigenicity by activating signal transducer and activator of transcription-3 (Stat3) and Sonic Hedgehog pathways in CSCs [39]. Cancer stem cells are known to play an important role in tumor growth, invasion, metastasis, and drug resistance in different types of tumors (Liu Y. et al., 2023; Lu et al., 2023; Sahara et al., 2023).

Clinical studies have revealed that patient survival time corresponded with the density, activation, and histological location of TAMs (Tang et al., 2013; Donadon et al., 2020). Targeting TAMs enlarges the antitumor effect of nanodrugs by secreting cytokines. Additionally, the pro-tumor activities mentioned above are driven by a specific subset of macrophages expressing canonical markers. Targeting these typical markers presents a viable approach to inhibit TAMs’ recruitment and survival, or alter their phenotypes effectively (Cassetta and Pollard, 2018). Therefore, TAMs are taken as ideal targets to influence tumor progression.

### 2.1 Nanomedical antitumor strategies by targeting TAMs

**FIGURE 1:**
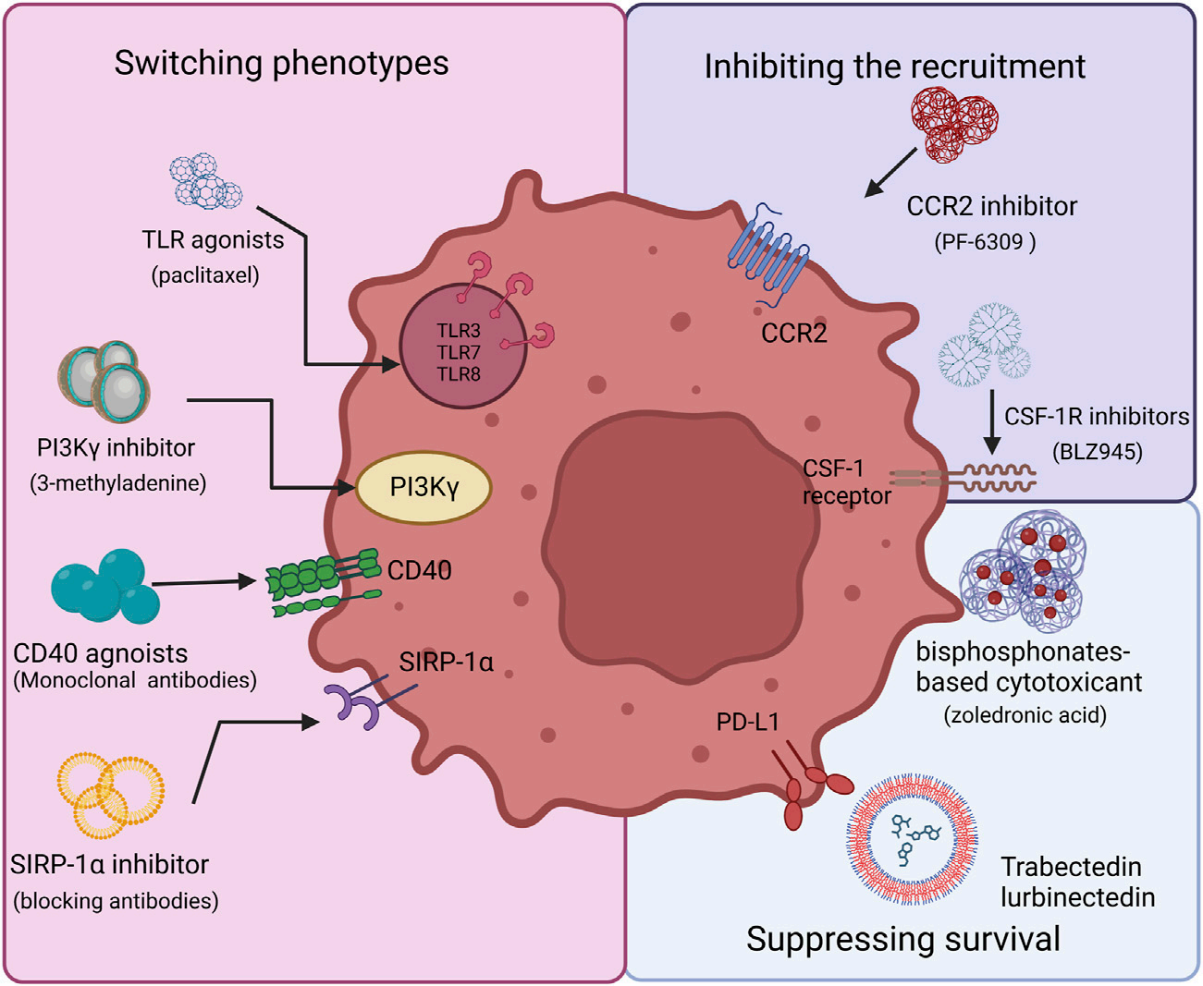
Therapeutic strategy options for TAM targeting. There are three main aspects to targeting TAMs, including inhibiting TAM recruitment, suppressing TAM survival, and switching TAM phenotypes. The CSF-1/CSF-1R and C-C Motif Chemokine Ligand 2 (CCL2)/C–C motif chemokine receptor 2 (CCR2) axis are required to recruit TAMs and related inhibitors, such as BLZ945, which could disrupt the process. TLR, PI3Kγ, CD40, and SIRP-1α play vital roles in reprogramming TAMs. Agonists to CD40 or Toll-like receptors activate TAMs and induce macrophage polarization. SIRP1α inhibitors prevent phagocytosis from being blocked. Via BTK–PI3Kγ signaling, selective inhibition of PI3Kγ can promote TAM reprogramming. Bisphosphonates show toxicity towards macrophages, which can be used to reduce their number.

To date, the main directions of research in this field include 1) inhibiting TAM recruitment, 2) inhibiting TAM survival, 3) switching M2 TAM into M1 phenotype to reduce pro-tumoral M2 macrophages and activate their antitumor functions. We list some of the latest laboratorial projects of TAM-targeted nanomedicines in Table 1. By precisely targeting the TAMs, nanomedicines can meet the demand for high concentrations of antitumor drugs and minimize potential threats to systemic macrophages, such as Kupffer cells in the liver (Biswas et al., 2013). In Figure 1, we showed the TAM targeting therapeutic strategies in this manuscript.

**TABLE 1:** Pre-clinical TAM-targeted nanomedicines in recent years.

Name	Target	Nanomaterial	Drug	Effect	Ref
Curcumin-Loaded Cationic Liposome-siRNA Complex	STAT3	Cationic liposomes	Anti-STAT3 siRNA	Re-education	Jose et al. (2018)
CDNP-R848	TLR7	β-Cyclodextrin	R848	Re-education	Rodell et al. (2018)
R848-loaded PLGA nanoparticles (PR848)	TLR7/8	PLGA	R848	Re-education	Wei et al. (2021a)
mannan conjugated Ce6 loaded with R848 (MCR)	TLR7/8	mannan polysaccharide, chlorin e6 (Ce6)	R848	Re-education	Uthaman et al. (2022)
CNT-CpG	TLR-9	CNT	CpG	Re-education	Fan et al. (2012)
GO-PEG-PEI	TLR-9	GO	CpG	Re-education	Tao et al. (2014)
Clarith/Kriketo/Azith Coated AuNPs	HSP70	Clarith, Kriketo, Azith Au	–	Re-education	Dreaden et al. (2012)
PHNPs@DPA-S-S-BSAMA@3-MA	PI3K	Hollow iron oxide	3-MA	Re-education	Li et al. (2020)
Nano-PI	PI3K	albumin	PTX, IPI-549	Re-education	Song et al. (2022)
miR497/TP-HENPs	PI3K	Exosomes, liposomes	miR497 TP	Re-education	Li et al. (2022)
cGAMP-NP	STING pathway	DOTAP; PC	cGAMP	Re-education	Cheng et al. (2018)
(HA-PEI)-microRNA 125b	CD44	HA; Poly ethylenimine (PEI)	miR-125b	Re-education	Parayath et al. (2018)
LMWHA-MPB NPs	CD44	HA; Mesoporous Prussian Blue (MPB	–	Re-education	Zhang et al. (2019)
mPEG-b-PHMA	CD44	HA; PLGA	SN38	Re-education	Huang et al. (2016)
Ab-(J-U)n	PD-L1	PD-L1 antibody; nanocarrier	TLR agonist	Re-education	Gong et al. (2023)
Dic@M2pep-Fe-MOF	—	Iron-based carrier; M2pep	Diclofenac	Re-education	Wei et al. (2022)
MAPEI -CpG	—	PEG;PLL;DMMA	CpG	Re-education	Du et al. (2023)
DOX@MAN-BSA	CD206	SA;mannose	Doxorubicin	Interference with survival	Zeng et al. (2022)
CSP-H8-D	σ-receptor	CSP; H8	Doxorubicin	Interference with survival	Jaggarapu et al. (2023)
Clol-Lipo-DOTAP	F4/80	liposome	Clodronate	Interference with survival	Piaggio et al. (2016)
PSNP	MMP2	PS; cholesterol; PEG2k-pp-PE	Diphosphonate	Interference with survival	Liu et al. (2020)
M2NPs-siRNA	—	α-peptide; M2pep	Anti-CSF-1R siRNA	Interference with survival	Qian et al. (2017)
Ethylene glyco-containing polymer-siRNA	—	Ethylene glyco-containing polymer	CXCL12 siRNA	Inhibition of recruitment	Wang and Wang (2023)
CX3CL1 siRNA deliver platform	CX3CL1 mRNA	7C1 nanoparticle	CX3CL1 siRNA	Inhibition of recruitment	Jung et al. (2017)
BisCCL2/5i mRNA	CCL2/5	liposome	BisCCL2/5i mRNA	Inhibition of recruitment	Wang et al. (2021b)
MiR-PCPmP	CD206	PEG; mannose	MiR155	Activate immune function in TME	Li et al. (2023)
Zn-CDA	—	Cyclic zinc; CDA	STING agonist	Activate immune function in TME	Yang et al. (2022b)
DS-Ce6	CD206 or F4/80	Dextran sulfate-based nano-photosensitizer	—	Photodynamic therapy	Park et al. (2022)
HA-BP	CD44	BP; HA	—	Photodynamic therapy	Zhang et al. (2021c)
Antibody-drug conjugate	CD47	SA; anti-CD47 (αCD47)	Photosensitizer IR820	Photodynamic therapy	Lu et al. (2022)

PEG, polyethylene glycol; PLL, poly-L-lysine; DMMA, dimethylmaleic anhydride; CSP, carbon nanosphere; PS, phosphatidylserine; SA, serum albumin; BP: black phosphorus.

#### 2.1.1 Inhibiting the recruitment of TAMs

##### 2.1.1.1 Suppressing the CCL2/CCR2 pathway

Many cells in TME were reported to secrete CCL2, such as monocytes, tumor cells, and stromal cells. As a chemokine, CCL2 interacts with its receptor CCR2 and plays a crucial role in bone marrow derived monocytes recruiting in solid tumors. These recruited monocytes then differentiate into TAMs (Argyle and Kitamura, 2018). Recent studies have provided compelling evidence that targeted inhibition of CCL2/CCR2 with specific drugs can effectively suppressed the recruitment of TAMs and impede tumor progression and metastasis in lung, liver, and breast cancer models (Bonapace et al., 2014; Li X. et al., 2017; Cane et al., 2019). There are two main exploration directions in CCL2/CCR2 blocking clinical trials, CCL2-blocking antibodies and small molecule inhibitors of CCR2.

One promising research area is the utilization of specific monoclonal antibodies to CCL2. Several monoclonal antibodies have been successfully developed, demonstrating significant potential in experimental settings by effectively reducing TAM infiltration and tumor progression (Horuk, 2009). As the first drug to enter clinical trials, carlumab (CNTO 888), a fully human immunoglobulin G1κ (Ig G1κ) specific to human CCL2, has shown its high affinity (15 p.m.) and remarkable effectiveness in blocking CCL2. During the experiment, advanced solid tumor patients were administered increasing doses of carlumab, leading to notable therapeutic outcomes. These outcomes encompassed effective inhibition of CCL2 levels within a short timeframe, considerable deceleration in the growth of primary tumors and distant metastatic sites, suppression of angiogenesis, and a subsequent reduction of microvessel density in preclinical models (Sandhu et al., 2013). However, the experimental data also revealed that treatment with carlumab alone could only achieve a transient inhibition of free CCL2 levels. The rapid escalation of dosage required for its effectiveness posed a challenge to its clinical implementation. This phenomenon was similarly observed in Irene Brana et al. research (Brana et al., 2015). Progress has been made in this treatment approach through the development of new monoclonal antibodies and exploring alternative drug administration methods, such as combination therapy with other antitumor drugs. However, there was still an issue of excessive drug concentrations in this approach (Cho et al., 2019).

Thus, targeted delivery of CCL2 blocking antibodies with the help of nanomedicines has receive more attentions. To manipulate the immune response mediated by CCL2, Yun Liu et al. developed a protein trap with a high affinity and specificity for CCL2, which was a CCL2-binding single-domain antibody. The plasmid DNA encoding this CCL2 trap, referred to as pCCL2, was delivered specifically to the TME using targeted lipid-protamine-DNA (LPD) nanoparticles. This localized delivery allowed for the expression of the CCL2 trap within the TME, aiming to improve immunosuppressive TME conditions (Liu et al., 2021). In comparison to the commercially available CCL2 antibody, this approach demonstrated improved therapeutic effectiveness and notable inhibition of tumor growth.

Blocking CCR2 using small molecule inhibitors is another research area in this field. Xiaoguang Li et al. reported that the blockade of CCL2/CCR2 signaling, achieved through CCR2 knockout or using a CCR2 antagonist, hindered the recruitment of inflammatory monocytes and prevented the infiltration and M2 polarization of tumor-associated macrophages (TAMs), leading to the reversal of immunosuppression within the tumor microenvironment. Moreover, this blockade also activated an effective inhibition of malignant growth and metastasis, reduced the risk of postsurgical recurrence and improved overall survival in hepatocellular carcinoma (HCC) patients (Li X. et al., 2017). This blockade of CCR2 has also shown certain therapeutic efficacy when used in combination with conventional chemotherapy drugs, such as the CCR2 inhibitor PF-04136309 combined with FOLFIRINOX chemotherapy (Nywening et al., 2016). This multi-drug combination therapy has been further enhanced and achieved with the assistance of nanocarriers. Zhuoya Wan et al. have developed a dual-delivery system that combined a STING agonist with a CCR2 inhibitor. It enabled effective activation of STING while simultaneously suppressed the immune resistance triggered by TAM recruitment. The study utilized a super-small-sized micelle system based on gemcitabine-conjugated polymer (PGEM), which exhibited excellent penetration ability in a pancreatic tumor spheroid model, and incorporated the CCR2 antagonist PF-6309 (PF) into the PGEM micelle system. The addition of CCR2 inhibitors effectively reduces the recruitment of TAMs and prevents immune suppression induced by PGEM, providing a new approach for maximizing the use of other relevant treatment strategies (Wan et al., 2023).

##### 2.1.1.2 Blocking the pathways of CSF-1R

Colony-stimulating factor-1 receptor (CSF-1R) belongs to the type III protein tyrosine kinase receptor family (Cannarile et al., 2017). The CSF-1R binding induces homodimerization of the receptor and activates receptor signals. Abnormal expression of CSF-1R plays a crucial role in the recruitment of TAMS. Meanwhile, blocking the CSF-1R-mediated signal pathway with an inhibitor has demonstrated great effect on the reduction of TAMs. Regulating the CSF-1/CSF-1R axis through receptor inhibitors can improve patient survival by controlling CSF-1 phosphorylation, which facilitates macrophage proliferation and conversion to TAMs (Lu and Meng, 2019). However, some CSF-1 R inhibitors have shown systemic safety concerns and poor tumor penetration. To reduce inhibitor toxicity to systemic macrophages, researchers have developed a series of nanocarriers for targeted drug delivery (Mun et al., 2020).

As a highly selective small molecule inhibitor of CSF-1R, BLZ945 inhibits the recruitment of TAMs through the blockade of CSF-1R signal way, while increasing the number of CD8 (+) T cells infiltrating tumors (Strachan et al., 2013). Therefore, BLZ945 was chosen by a number of researchers to develop an antitumor treatment. Liaw et al. developed a novel dendrimer conjugate for BLZ945 targeted delivery. Through modification by succinic anhydride, the cyclohexane ring in BLZ945 carried a pH- and esterase-sensitive linker in 90% yield. The linker had a carboxylic acid terminal group for covalent connections to the surface of the polyamidoamine (PAMAM) dendrimer, which had a high density of hydroxyl groups. By competitively binding to the receptor ATP binding site, the cyclohexanol carried by the platform could block phosphorylation to inhibit the recruitment of TAMs. This nanodrug delivery platform exhibited high safety and stability in intratumor conditions through a pH-sensitive linker and graded release. High Performance Liquid Chromatography (HPLC) analysis confirmed that BLZ was released as a free drug instead of in combination with a succinic linker. The release ratio of BLZ945 reached 42.7% in acidic pH simulating conditions after 18 h, far more than 16.3% in pH 7.4 conditions. These two points together ensured that BLZ945 had better tumor penetration (Liaw et al., 2021).

In addition to focusing on pH-induced drug release, Yuchi Wang et al. has also enhanced the concealment ability of BLZ945 loading nanoparticles. They designed a pH-responsive copolymer micelle, camouflaged with a hybrid membrane of erythrocytes and cancer cells. It was developed for targeted delivery of BLZ945 to TAMs. Initially, a dextran-g-poly (histidine) copolymer was synthesized, and BLZ945 was incorporated into the micelles through hydrophobic interaction, forming DH micelles. Subsequently, the DH micelles were coated with a hybrid membrane of erythrocytes and cancer cells (ECm) to create the final DH@ECm formulation. When intravenously administered into the bloodstream, DH@ECm exhibited prolonged circulation time and immune camouflage properties. It has been demonstrated to effectively accumulate in the tumor area and thereby inhibit the recruitment of M2-type macrophages (Wang et al., 2020).

In contrast to the single nanoparticle strategy, the multi-nanoparticle combination strategy can also effectively reduce the accumulation of M2-type macrophages in the tumor. Qi Wei et al. developed two types of nanoparticles, including Combretastatin A4 nanoparticles (CA4-NPs) and FXIIIa substrate peptide A15 decorated BLZ945 nanoparticles (A15-BLZ-NPs). CA4-NPs accumulated in tumor vessels will lead to tumor hemorrhage and coagulation reactions which enabled the circulating A15-BLZ-NPs to selectively target the CA4-NP- accumulated tumors through coagulation targeting. The combination treatment resulted in a 3.75-fold increase in the distribution of BLZ945 in the tumor, effectively inhibiting the enrichment of M2-type macrophages (Wei et al., 2020).

Apart from BLZ945, pexidartinib (PLX)-based nanomedicines also show efficiency in inhibiting the recruitment of TAMs. Zhaoting Li et al. have developed a biocompatible alginate-based hydrogel that encapsulated nanoparticles loaded with PLX. This hydrogel gradually released PLX at the tumor site to block colony-stimulating factor 1 receptor (CSF1R) and reduce TAM density. This approach not only prevented the recruitment of TAMs but also reactivated T cells to enhance the immunotherapeutic effect against tumor recurrence (Li et al., 2022). Pang, L et al. used M2pep to modify the polymeric nanoparticles and PLX encapsulated in M2pep-coated nanoparticles attenuated the M2-type macrophages (Pang et al., 2019).

##### 2.1.1.3 Inhibiting the function of VEGF

During the development of tumor, VEGF plays a role in pro-angiogenesis, which creates a favorable microenvironment and promotes tumor growth. (Elebiyo et al., 2022). In solid tumors, circulating monocytes can be recruited by many tumors-derived chemical inducers including macrophage chemoattractant protein (MCP)-1 and VEGF (Ueno et al., 2000). Subsequently, TAMs stimulate angiogenesis and further recruitment of TAMs by releasing potent pro-angiogenic cytokines and growth factors, including VEGF-A, bFGF, TNF-a, and IL-8 (Gerber et al., 2010). Therefore, the effective inhibition of TAM recruitment can be achieved by reducing the levels of VEGF in the TME through pharmacological interventions. Some current studies have also validated the potential research value of this approach, including through some anti-VEGF antibodies like bevacizumab or pan-VEGFR tyrosine kinase inhibitor to block VEGF (Peterson et al., 2016; Deng et al., 2017).

TAMs are equipped with many specific receptors on their surface, and targeting TAMs directly with modified nanocarriers to transport anti-VEGF drugs is a common therapeutic approach. For instance, the mannose receptor (MR) is a highly effective endocytic receptor that overexpresses on the surface of TAMs and mannose modification is an efficient targeting strategy in the design of nanoparticles (NPs) (Xiong et al., 2017). Regorafenib (RG), a promising oral multikinase inhibitor, has demonstrated potent inhibition of endothelial cell kinases in both biochemical and cellular kinase phosphorylation assays (Wilhelm et al., 2011). Bai et al. developed mannose-modified γ-cyclodextrin (M-γ-CD) with RG, RG@M-γ-CD NPs, which showed high-order self-assembly. Apart from the previous CD-based nanomedicine, this modification induced the formation of channel structures (channel-type nanoparticles, CNPs) while enabling vectors to target TAMs. The noncovalent channel structure optimized the distribution and pharmaceutical properties of RG in tumor tissue, which regulated the VEGF/VEGFR axis by blocking tyrosine kinase with immunoglobulin. Compared with control groups treated with RG, the RG@M-γ-CD group significantly inhibited the secretion of VEGF and lesion neovascularization, which decreased the recruitment of TAMs in tumor tissue (Bai et al., 2021). Furthermore, Yudong Song et al. used polyethylene glycol (PEG) and mannose doubly modified trimethyl chitosan (PEG = MT) to develop NPs, which was along with citraconic anhydride grafted poly (PC) to form PEG = MT/PC NPs. The novel NP was dual pH-responsiveness and the modification of PEG further enhanced the targeting ability of NPs based on mannose, which can carry VEGF siRNA (siVEGF)/PIGF siRNA (siPIGF) to M2-TAMs. It simultaneously caused efficient gene silencing of VEGF and PIGF and reduced macrophage recruitment (Song et al., 2018).

However, considering that the mannose receptor is also expressed in normal epithelial cells and dendritic cells to some extent, some researchers choose to use peptide sequences with higher specificity to target TAMs (Cieslewicz et al., 2013). João Conde et al. developed RNAi-M2pep-AuNPs which were consisted of a 15 nm gold core decorated with a thiolated-polyethylene glycol (PEG)-COOH polymer and further functionalized with TAM targeted M2pep. The specific anti-VEGF siRNA combined RNAi-M2pep-AuNPs have proven to be effective in addressing the challenges of limited therapeutic targets and possible off-target effects, and result to improved treatment outcomes. Along with silencing angiogenic factors, the VEGFi-M2pep-AuNPs inhibied the recruitment of macrophages in lung tissue and lung lavage, indicating their specificity in targeting TAMs (Conde et al., 2015).

Apart from directly targeting TAMs to interfere with VEGF secretion, regulating the hypoxia environment in TME to indirectly control the expression level of VEGF is also a viable therapeutic strategy. The correlation between the number of TAMs and intratumorally hypoxia provides strong evidence supporting the stimulatory effect of hypoxia on the recruitment of macrophages into the hypoxic regions of neoplastic tissue (Osinsky et al., 2011). Hence, employing pharmaceutical interventions to ameliorate the hypoxic conditions and remodel TME can effectively inhibit the secretion of VEGF and diminish the recruitment of TAMs. Manli Song et al. designed HA-modified MnO2 nanoparticles (Man-HA-MnO2 NPs), which were targetedly delivered to TAMs. And under hypoxic conditions, they significantly improved tumor oxygenation levels by reacting with endogenous H2O2. This led to the downregulation of hypoxia-inducible factor-1α (HIF-1α) and VEGF levels in the tumor. Consequently, the migration of TAMs to the TME was effectively inhibited, simultaneously suppressing angiogenesis and TAM recruitment (Song et al., 2016).

#### 2.1.2 Suppressing survival of TAMs

In addition to reducing the recruitment of TAMs in tumor, eliminating TAMs already present can also slow the development and progression of the tumor. In recent studies, several strategies have been shown to directly induce the apoptosis of TAMs. Here, we mainly focus on TAM-targeting nanoparticles based on cytotoxic drugs and some other functional NPs.

##### 2.1.2.1 Cytotoxic drugs -based nanomedicine

Toxic drugs commonly used in clinical practice including bisphosphonates and trabectedin have been demonstrated to possess the ability to effectively reduce macrophages through the induction of their apoptosis.

Bisphosphonates are anti-resorptive drugs that act on osteoclasts to inhibit bone resorption and bone metastasis, such as zoledronic acid (ZA) and clodronate (Zhu et al., 2013). Macrophages are with high phagocytic ratio to bisphosphonates, which leads to TAMs’ functional impairment and depletion (Rogers and Holen, 2011; Junankar et al., 2015). However, the application of bisphosphonates may cause damage to residential macrophages in normal tissues. This makes the technical research of relevant nanocarriers particularly important.

The application of liposomes in the early stage has achieved some curative effects. Hattori et al. conducted a trial of ZA and liposomal doxorubicin (Doxil^®^) combination therapy. When ZA solution was injected at 40 µg/mouse per day for three consecutive days into mice bearing murine Lewis lung carcinoma LLC tumor, TAM depletion was promoted, and angiogenesis at the tumor site was significantly inhibited. This was based on the improved distribution of the drug within the tumor, which could not be observed in ZA treatments alone (Hattori et al., 2015). Considering the broad impact of liposomes on the systemic mononuclear-macrophage system, the concept of fine-tuning their size for enhanced drug delivery has emerged as a potential strategy to mitigate systemic toxicity. For instance, Piaggio, F et al. developed a novel Clodronate-containing liposomes with cationic lipid (2,3-Dioleoyloxy-propyl)-trimethylammonium (DOTAP), called Clo-Lipo-DOTAP. Through the evaluation of nanoparticles with size gradients, they discovered that liposomes with a diameter of 200 nm demonstrated superior TAM targeting. These smaller, spatially stable liposomes exhibited enhanced TAM specificity without causing hepatotoxicity or nephrotoxicity, which were utilized to deplete TAMs s in primary murine melanoma models (Piaggio et al., 2016).

Chemical modification can also improve the targeting ability of liposomes, Xueying Tang et al. have developed a novel nano-drug delivery platform by formulating ZA liposomes and modifying them with an SA-octadecyl amine conjugate (ZA-SL). This platform enabled the effective delivery of ZA into TAMs through the binding of SA to Siglec-1, resulting in specific cytotoxicity towards M2 TAMs in a concentration-dependent manner. The authors have modified the structure of Sialic acid (SA) through amide coupling with octadecyl amine (ODA) using EDCHCl/NHS as a catalyst, yielding an SA-ODA conjugate that exposed the SA moiety on the surface of the liposomes. This exposed SA moiety could be recognized by receptors on TAMs, enhancing internalization and targeted drug delivery to TAMs. Cell toxicity analysis revealed that the polar nature of ZA solution almost had no inhibitory effect on cells, while the cell survival rate in the ZA-SL group was significantly lower than that in the ZA group (*p* < 0.01), indicating that ZA-SL exhibited high specificity and strong cytotoxicity towards TAMs (Tang et al., 2020). Considering the general toxicity of bisphosphonates on mononuclear-macrophage system, the exploration of alternative nanocarriers is an important focus in this field. Wan, X et al. developed a red blood cell (RBC)-derived nanovesicles (RDNVs) based nano platform for effective hydrophilic drug delivery. Compared to clodronate-encapsulated liposomes, CD47 KO mouse RBCs (KO-RDNVs) encapsulated with clodronate showed more biocompatible and less toxic, which provided efficient and long-term macrophage depletion (Wan et al., 2019).

Furthermore, trabectedin is regarded as an anti-neoplastic drug, which induces cell apoptosis through a TNF-related apoptosis-inducing ligand (TRAIL)-dependent pathway. Based on the expression of functional TRAIL receptors on monocytes and macrophages, Trabectedin shows a specific cytotoxic effect on TAMs (Germano et al., 2013). Morton s et al. developed a TAM-targeting liposome containing trabectedin, which comprised an antibody or fragment selectively binding to TAM-specific clearance agent receptor CD163. By inducing the rapid and selective apoptosis of monocytes and macrophages, it improved the immunosuppressive environment and rescued the functional activity of T cells (Morton and Solinas, 2023).

##### 2.1.2.2 Functional Nanoparticles

It has been reported that the binding of photothermal therapy (PTT) with nanoparticles can effectively deplete TAMs.

Indocyanine green (ICG) is a registered NIR fluorescence-imaging agent, which is a superfine photothermal agent candidate. The combination with nanoparticles can solve the obstacles to its stability and tumor targeting. Dong-Hua Wan et al. developed a novel noncovalent ICG conjugate of C-phycocyanin (CPC) (ICG@CPC). As a TAMs targeted vehicle, CPC increased threefold photothermal conversion efficiency, compared with free ICG and improved NPs’ stabilities. ICG@CPC could specifically target TAMs to inhibit the activity of TAMs under 808 nm laser irradiation (Wan et al., 2020). On this basis, Gao, S et al. proposed combined therapy of PTT and photodynamic therapy (PDT). They developed a pH-sensitive mesoporous calcium silicate nanocomposite (MCNs), which was encapsulated with ICG. Under the 808 nm near-infrared (NIR) light, the nanocarrier could trigger PTT and PDT, inducing apoptosis of TAMs through intracellular singlet oxygen in the cell (Gao et al., 2022).

Besides, magnetic heating therapy has also shown potential research value. The team led by Wang et al. successfully synthesized a novel type of Magnetic-Fe/Fe3O4 nanoparticle. By modifying the surface of the nanoparticle with a detachable carboxylesterase linkage, they were able to integrate an antitumor drug, SN38, into the nanoparticle. The study demonstrated that treatment with SN38-NPs and doxycycline induced the expression of carboxylesterase in RAW264.7 cells, which facilitated the detachment of SN38 from the core/shell Fe/Fe3O4 nanoparticles. This combination of the magnetocaloric effect provided by the magnetic nanoparticle and the therapeutic efficacy of the released SN38 resulted in a remarkably potent anti-cancer effect (Wang et al., 2012).

#### 2.1.3 Switching of TAM phenotype

In the presence of TME signals, a significant portion of TAMs undergo activation and adopt an M2 (alternatively activated) phenotype. Related studies have proven that M2 macrophages play a vital role in the development of tumor tissue, including tumor cell proliferation, tumor angiogenesis, and systemic immunosuppression (Boutilier and Elsawa, 2021). On the contrary, as classic polarization, M1 macrophages can express the corresponding proinflammatory factors TNF-α, IL-12, and iNOS to clear pathogenic microorganisms. Signaling pathways associated with TAM polarization include phosphoinositide 3-kinase (PI3K)/Akt pathway, TLR7/8, which is involved in the polarization, survival, growth, proliferation, differentiation, and apoptosis of M2-like TAMs (Yang D. P. et al., 2022). Indirectly inducing the polarization of M2-like TAMs by modulating the TME is also a strategy. In light of these existing research directions, we mainly summarize the relevant studies on nanomedicine targeting at PI3Kγ, Toll-like receptors etc.

##### 2.1.3.1 Regulating the function of PI3Kγ

During inflammation, PI3Kγn is involved in a major switch between immune stimulation and suppression (Kaneda et al., 2016). Through Akt and mTOR, PI3Kγ inhibits the activation of NFκB and stimulates the revitalization of C/EBPβ, activates transcriptional programs which promote tumor development. Regulating PI3K/Akt axis is an important way to participate in M2-like TAMs polarization, survival, growth, proliferation and apoptosis (Marquard and Jucker, 2020; Yang D. P. et al., 2022). Consequently, targeted inhibition of the PI3K pathway in TAMs can effectively promote the polarization of M2-like TAMs and disrupt the immunosuppressive environment.

In recent years, significant progress has been made in the research of targeted delivery of small molecule inhibitors of PI3K, along with other drugs, through nanocarriers. Unlike previous single-molecule drug delivery, Li et al. developed a novel PHNP@DPA-S-S-BSA-MA@3-MA system as the combination therapy with PHNPs (Porous Hollow iron oxide Nanoparticles)and 3-MA (3-methyladenine, a small molecule inhibitor of PI3K γ) (Li et al., 2020). This system efficiently suppressed PI3K γ protein expression, which played a vital role in the transformation of M2 TAMs into the M1 type. The targeting of this nano platform relied on surface modification of carbonylated mannose, which has demonstrated excellent targeting of M2 macrophages in a large number of experiments. What’s more, compared with other mannose-modified nanoparticles, the introduction of bovine serum albumin (BSA) blocked 3-methyladenine, which ensured that the drug would not be released prematurely (Li et al., 2020). The efficacy of combination therapy has also been demonstrated in other studies. Song, YD et al. combined paclitaxel (PTX) and immunomodulators PI3K beta inhibitor (IPI-549) in an albumin nanoparticle, Nano-PI. Nano-PI in combination with α-PD1 can not only reshaped the immune microenvironment to induce M2 macrophage polarization towards the M1 phenotype, but also increased the population of CD4 (+) and CD8 (+) T cells, B cells and dendritic cells while suppressing regulatory T cells to prevent T cell exhaustion (Song et al., 2022).

With a focus on exploiting homotargeting properties, Longxia Li et al. developed a novel bioinspired hybrid nanoplatform by combining CD47-expressing tumor exosomes with cRGD-modified liposomes. The homotargeting properties of tumor exosomes enhanced their TAMs-targeting and retention capabilities. cRGD-modification helped miR497 and triptolide (TP) release in the acidic tumor, which upregulated the polarization of macrophages from M2 to M1 macrophages by inhibiting the PI3K/AKT/mTOR signaling pathway (Li Z T et al., 2022).

##### 2.1.3.2 Targeting Toll-like receptors

Toll-like receptors (TLRs), as pattern recognition receptors of the innate immune system, can activate macrophages and induce an M1-like functional polarization when stimulated by their ligands (Mantovani et al., 2017). Research on nanomedicines targeting receptors including TLR4 and TLR7/8 has made significant progress.

In light of TLR7/8, researchers have developed special nanocarriers for specific drugs to achieve targeted TLR7/8 agonist transportation. Synthetic adjuvants based on small molecule TLR7/8 agonists encompass imidazoquinolines (IMQs) and benzazepines. Among these, resiquimod (R848) stands out as one of the most extensively investigated IMQs to date (Dowling, 2018). Rodell, CB et al. chose to utilize β-cyclodextrin (CD) as a supramolecular drug reservoir, which forms cyclodextrin nanoparticles (CDNPs) with macrophage affinity and high drug-loading capacity. This nanocarrier, while maintaining mechanical properties and TAM-associated distribution, could carry a larger amount of R848, an agonist of TLR7 and TLR8, to promote the conversion of the M2 phenotype (Rodell et al., 2018). Besides, progress has been made in researching drug safety and biocompatibility. Wei et al. developed a nanoparticle-loaded bacterial system Ec-PR848, which recruited at the tumor site through E. coli-induced hypoxia targeting. R848 was packaged by PLGA to prepare R848-loaded PLGA nanoparticles (PR848). The innovations of the system fixed PR848 on the surface of *E. coli* by electrostatic force, which made good use of the biological properties of *Escherichia coli* and improved biosafety compatibility. This nanosystem had excellent conversion efficiency with an M1/M2 ratio of 1.34 (Wei B. C. et al., 2021). The addition of photodynamic therapy to conventional chemotherapy can, to some extent, improve drug distribution. Uthaman, S et al. chose to synthesize a novel synergistic immune-photodynamic nanocarrier. This carrier involved the conjugation of TAM-targeting mannan polysaccharide with chlorin e6 (Ce6) photosensitizer, followed by loading with R848. As a bioconjugated nanoparticle, this carrier selectively targeted anti-inflammatory M2-like cells. In addition, through photodynamic therapy, it released the stimulant to repolarize the anti-inflammatory M2-like cells into pro-inflammatory M1-like cells (Uthaman et al., 2022).

Similarly, some studies have revealed the positive effect of TLR4 on TAM polarization. To evaluate the immune effect of TLR4, Wanderley et al. treated melanoma-bearing mice with paclitaxel, which significantly reduced the number of TAMs positive for CD206 (an M2 marker). However, this experimental result cannot be reproduced in TLR4-deficient mice, which suggests that the immune mechanism of paclitaxel involves TAM polarization via TLR4 activation (Wanderley et al., 2018). The critical role of TLR4 was similarly confirmed by the effects of other nanoparticles. GDNPs, a novel EV-like ginseng-derived nanoparticle isolated by Cao, M., et al., dramatically promoted the polarization of the M2 to M1 phenotype. The response induced by GDNPs on macrophages is similar in the TLR4/MyD88 (myeloid differentiation antigen 88) pathway induced by pathogen-associated molecular patterns (PAMPs) (Cao et al., 2019).

##### 2.1.3.3 Remodeling the tumor microenvironment

Malignant cells, including tumor cells and M2-type tumor-associated macrophages (TAMs), contribute to the construction of the TME by secreting substances like VEGF, platelet-derived growth factor (PDGF) and IL-10. This process promotes the polarization of macrophages toward the M2 phenotype, ultimately. Reshaping the TME holds significant implications in facilitating the transition of TAMs from M2 to M1 phenotype (Boutilier and Elsawa, 2021).

By combining polarization-inducing agents and immune stimulatory drugs, it is possible to effectively overcome immune suppression barriers, promote the reprogramming of TAMs from M2 to M1 phenotype, and simultaneously reshape the TME. In the majority of previous studies, CpG-DNA has been widely employed as a common polarization-inducing agent to induce a phenotypic transformation in TAMs (Yuan et al., 2017). Shulan Han et al. developed a PLGA nanoparticle encapsulating baicalin and CpG oligonucleotide (CpG-ODN), which was coated with polydopamine, M2pep and α-peptide for specific targeting and ligand attachment (Han et al., 2021). Baicalin was demonstrated by Gong et al. that could independently stimulate the proliferation of T and B cells, as well as synergistically with concanavalin A (Con-A) or lipopolysaccharide (LPS) (Gong et al., 2011). The nanoparticle successfully targeted M2-like TAMs *in vitro* and *in vivo* studies, inducing a phenotypic shift from M2-like to M1-like which remodeled the TME. Activated M1-like macrophages and T cells release certain cytokines such as IL-2, IL-12, TNF-α, and IFN-γ, which decrease the secretion of VEGF at the tumor site. This further transforms the TME into an anti-tumor microenvironment, facilitating the conversion of TAMs (Gong et al., 2011).

Similarly, experimental evidence suggests that iron nanoparticles enriched in the tumor vicinity can indirectly alter the (TME to promote the polarization of TAMs (Zanganeh et al., 2016). In order to enhance the reprogramming capability and augment the antitumor effect based on the conventional ferumoxytol nanoparticles, Wu et al. conducted a study where hollow Fe3O4 NPs were loaded with L-arginine (L-Arg) and sealed using poly (acrylic acid) (PAA). This led to the development of LPFe3O4 NPs, which exhibited a pH-responsive behavior, enabling controlled release of L-Arg. The main objective of this reprogramming approach was to induce the release of proinflammatory cytokines and attract T cells, thereby facilitating the elimination of tumor cells. Moreover, the LPFe3O4 NPs facilitated the production of nitric oxide (NO) through the overexpression of nitric oxide synthase (iNOS) by M1 TAMs. The resulting NO played a significant role in the elimination of tumor cells through gas therapy (Wu et al., 2021).

### 2.2 Magic bullets to target TAMs

**FIGURE 2:**
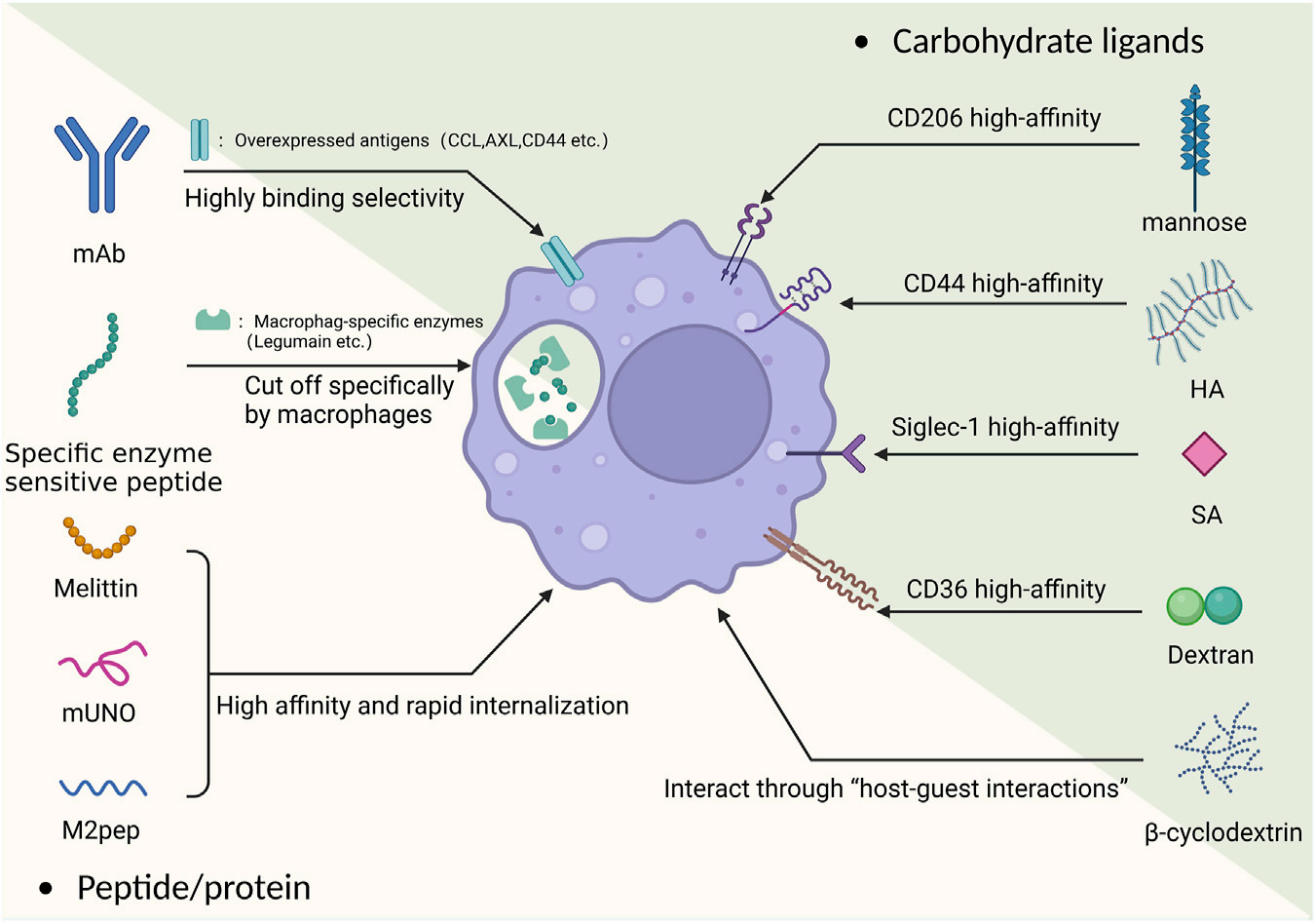
The basic principle of the “magic bullet” and TAM targeting carbohydrate and peptide/protein ligands.

The term “magic bullet” was first coined by Paul Ehrlich in the last century and was originally used to describe a drug that killed a parasite in a human host without harming the host itself (Wittlin and Mäser, 2021). Broadly speaking, this theory can be used for all human diseases as magic bullets can increase the drug’s targeting specificity and reduce side effects and off-target (Strebhardt and Ullrich, 2008). TAMs possess a number of specific overexpressed surface receptors, such as CD44, CD68, CD163, CD206, and CD204, which makes it possible for magic bullets to act on TAMs (Singh et al., 2017). Here, we concentrate on the modifications of the nanomaterials that promote their specificity and antitumor effect (Figure 2).

#### 2.2.1 Carbohydrate ligands

**The CD206 receptor** is an overexpressed mannose receptor on M2 macrophages. Targeting the CD206 receptor with mannose polymer-modified nanoparticles makes it possible to achieve targeted delivery on TAMs (such as delivering doxorubicin) (Niu et al., 2014). However, even the simple use of mannose-modified NP can achieve good targeting effects, for example, Zeng et al. recently used mannose-modified serum albumin (MAN-BSA) carrier to carry doxorubicin, which could achieve good results in killing both TAM and tumor cells simultaneously (Zeng et al., 2022). Previous studies still have shown that the affinity between mannose and CD206 is not ideal. On this basis, a recent study reported a new strategy for the targeted delivery of siRNA to macrophages and DCs using a high-affinity CD206 ligand-conjugated siRNA. Specifically, this ligand was modified with a “mannose ligand-conjugated tetravalent linker,” which could significantly improve its affinity and provide ideas for better targeting of M2 (Uehara et al., 2022). In a recent study, the improvement of the arrangement of mannose on the NP surface was applied. Ghitman et al. modified it using an O-palmitoyl-mannose interval arrangement method and measured its physical properties and targeting ability. The results showed that the accumulation of macrophages in the target area increased by two-fold, and the drug showed good encapsulation efficiency (Ghitman et al., 2022). The use of multiple ligand joint targeting is also one of the improvement ideas. Using the common mannose as a ligand, Song et al. developed a means of targeted delivery of manganese dioxide by coupling manganese dioxide particles with mannose polysaccharides and encapsulating them with hyaluronic acid (HA). Manganese dioxide can promote the decomposition of hydrogen peroxide, and the use of nanoscale manganese dioxide particles can reverse the hypoxic tumor microenvironment to some extent, transforming M2 macrophages into M1 macrophages (Song et al., 2016). In recent research, Chen et al. developed a dual-targeting nanoparticle that combined the originally targeted hyaluronic acid palladium nanoclusters (Pd-HA) with mannose and the imiquimod molecule (R837) to enhance macrophage targeting while reducing the number of cancer cells using Pd chemical drive therapy (Chen et al., 2022). Apart from co-ligands, the combination of the mannose and the carrier is also important. Uthaman et al. innovatively coupled TAM self-targeting acrylic acid grafted mannan with chlorin e6 (Ce6) and loaded R848 to achieve targeted reprogramming of M2 (Uthaman et al., 2022). In addition, the use of peptides to target CD206 receptors is also a new idea, which will be described below.

**Hyaluronic acid (HA)** can specifically bind to the overexpressed CD44 receptor on TAMs (Misra et al., 2015). It has a high biocompatibility and high effectivity and can be targeted with a variety of payloads. In pancreatic and ovarian cancer, Parayath et al. used HA-polyethyleneimine (HA-PEI) encapsulating miR-125b (a miRNA that regulated macrophage polarization) to reprogram TAMs into an antitumor (M1) phenotype (Parayath et al., 2019; Parayath et al., 2021). Wang et al. used their modified liposome-protamine-hyaluronic acid (LPH) nanoparticle to deliver anti-CD47 siRNA to achieve macrophage reprogramming (Wang et al., 2013).

At the same time, HA could be used to optimize existing nanomedicine therapies. Previous studies have shown that iron oxide nanoparticles could inhibit tumor growth by switching M2 to M1, but their toxicity cannot be ignored (Zanganeh et al., 2016). To optimize the existing nanoscale Fe3O4 loaded with CpG liposomes (Lipo), He et al. used mannosylated carboxymethyl chitosan and HA to modify nanoparticles, resulting in stronger targeting and lower toxicity (He et al., 2017). Meanwhile, in a recent study, Nie et al. were able to used mannose to confer TAM targeting ability, while HA and iron oxide were used to repolarize M2-like TAM. The NPs developed by Nie et al. significantly improved the targeting success rate and the cure rate in mice (Nie et al., 2022).

In the field of photodynamic therapy, nanodrugs need to be combined with photothermal or photodynamic effects to function, which requires certain stability, biocompatibility, and concentrated distribution of nanodrugs. The use of HA can meet the needs of photodynamic therapy well. Zhang et al. designed a targeted multifunctional black phosphorus (BP) nanoparticle modified with polyethylene glycol hyaluronic acid (HA). Fluorescence and photoacoustic imaging showed that it actively accumulated at the tumor site through CD44+-mediated active targeting, significantly improving the accuracy of the therapy (Zhang X. et al., 2021).

**Folic acid** (FA) is a water-soluble cellulose, and its receptor (FOLR) has been widely used as a tumor marker for targeted transport. It is divided into two subtypes (FOLR1 and FOLR2), of which FOLR2 has been shown to be overexpressed in TAMs (Kurahara et al., 2012). Using this feature, nanoparticles with various payloads can obtain the “magic bullet” effect. Together with other ligands, folic acid can enhance the target efficacy of antitumor-drug-loaded nanoparticles.

Pearl Moharil et al. used ultrasmall-sized gemcitabine (GEM)-based NPs coupled with FA to achieve a combination of passive and active targeting while simultaneously targeting both tumor cells and TAMs. On this basis, the team developed a GEM-based FA-modified drug carrier and loaded it with doxorubicin (DOX). Experiments confirmed that after systemic administration of the drug in a mouse breast tumor model, the concentration at the tumor site increased sharply compared with other organs and effectively inhibited tumor growth (Moharil et al., 2022). Using DOX as the payload, Xiong et al. employed a new dual-ligand co-targeting approach and a new nanocarrier, by chemically modifying FA and stearic acid onto rhodiola rosea polysaccharides nanoparticles (RHPs). This has significantly improved the encapsulation of the hydrophobic drug DOX. Experimental results showed that the delivery efficiency and the ability to regulate macrophage polarization were both enhanced by using this co-modified nanoparticle (Xiong et al., 2023). Furthermore, functional siRNA drug systems based on FA were effectively validated. For instance, Chen et al. used folic acid-conjugated carboxymethyl chitosan-coated nanoparticles to deliver STAT3 inhibitory siRNA for targeting and inhibiting M2. Experimental results showed that the inhibition effect and targeting accuracy of this method were significantly improved compared to normal delivery (Chen et al., 2020).

**Sialic acid (SA)** is a widely occurring carbohydrate, and its high-affinity receptor Siglec-1 has been found to be overexpressed on TAMs (Cassetta et al., 2019). Simultaneously, as an endocytosis-guiding receptor, Siglec-1 is very suitable as a targeting target. Qiu et al. took advantage of this feature to develop a new type of nanocarrier using a SA modified amphiphilic egg phosphatidylglycerol (EPG) structure to carry ibrutinib (IBR). Therefore, the IBR originally easily filtered by the kidneys could accumulate at the TAM-rich site, enhance its effect, and greatly reduce off-target and IBR side effects (Qiu et al., 2019). Similarly, a study used sialic acid-modified cyclodextrin nanocarriers to achieve the goal of overcoming the complex TME environment in prostate cancer, specifically delivering siRNA to TAMs, causing their reprogramming (Sun et al., 2023). At the same time, a new SA-based co-targeting approach was developed to ulteriorly strengthen the targeting ability. Ding et al. and Tang et al. developed targeted carriers based on SA-18 amine conjugate respectively loaded with epirubicin (EPI) and Zoledronic acid (ZA) to form nanoparticles EPI-sal and ZA-ODA respectively. ZA-ODA was proven to have good targeting ability and no significant side effects in mouse experiments (Tang et al., 2020), while EPI-sal’s pharmacological experiments showed that its anti-tumor effect was better than other groups (Ding et al., 2019).

**Dextran(polysaccharide)** is a typical ligand of the scavenger receptor CD36 on macrophages. The characteristic of CD36 is low expression on M1 macrophages but overexpression on M2 macrophages. The difference in distribution makes it promising as a targeting ligand (Liu and Roche, 2015). Yuan et al. developed dextran-modified polystyrene nanoparticles (DEX-PS) and studied their distribution after administration in mice. The results showed that they accumulated well in the TME but also aggregated at macrophage-related inflammatory sites (Yuan et al., 2020). Dextran has demonstrated its targeting value in the field of imaging. In the field of radiology, dextran has proven its targeting value. The metabolizable dextran-indocyanine green (DN-ICG) nanoprobe developed by Luo et al. can increase the fluorescence intensity nearly three times based on ICG, and solve the liver damage caused by the retention of fluorescent agents (Luo et al., 2021). On this basis, dextran can also be modified. For example, Zhou et al. used sulfated dextran to more effectively target the SR-A receptor of M2 macrophages and used it to coat nano-sized iron oxide particles for MRI imaging (Zhou et al., 2022).

**β-Cyclodextrin (CD)** has a similar chemical composition to linear polysaccharides and can interact with macrophages through host-guest interactions. There have been examples of its combination with nanoliposomes to utilize macrophages for the treatment of atherosclerosis (Rodell et al., 2018). Shi et al. found that CDNP-R848, formed by complexing resiquimod (R848, a TLR agonist) with CD, could also effectively target TAMs and induce macrophage reprogramming (Shi and Gu, 2021). The approach of using CDNP has been effectively validated in recent years. For example, in a recent experiment using CDNP to treat glioblastoma by Turco et al., very satisfactory results were achieved. It was demonstrated that CDNP-R848 induced tumor regression of experimental glioblastoma by targeting blood-derived macrophages, without the need for adaptive immunity (Turco et al., 2023). Currently, only a few drugs carrying TLR agonists have used this optimization scheme, so there is some potential in this direction.

#### 2.2.2 Peptide/protein

In addition to common carbohydrates, M2 macrophages also have a high affinity for a variety of peptides and proteins. Whether used as ligands or as carriers themselves, they can achieve the effect of a magic bullet.

**The M2 macrophage-targeting peptide mUNO** has become a new optimization approach in targeting the classical target CD206. The tumor-homing peptide (mUNO, sequence: “CSPGAK”) has been proven to be a novel CD206 ligand, which specifically binds to the target through electrostatic interactions, aromatic interactions, and hydrogen bonds. Studies have shown that fluorescein modified -mUNO (FAM-mUNO) has high selectivity for macrophages (Lepland et al., 2020; Lepland et al., 2022).

In order to attach it with less toxicity and better biocompatibility. Several relevant studies have been carried out. Firstly, the research team has developed OximUNO by combining the peptide with star-shaped polyglutamic acid (St-PGA). Experiments have shown that OximUNO had no acute liver or kidney toxicity in the body, and treatment with OximUNO has reduced the progression of primary tumor lesions and lung metastasis, significantly reducing the number of CD206+ TAMs (Lepland et al., 2022). In another study, mUNO was linked to a biocompatible lignin polymer carrier (LNPs) and loaded with a dual agonist of the toll-like receptors TLR7/8 (resiquimod, R848) to achieve targeted reprogramming of macrophages. The feasibility of this approach was demonstrated and its application prospects in cancers such as breast cancer were described (Figueiredo et al., 2021).

**Melittin (MEL)** has also been shown to have a high affinity for CD206-positive TAMs (Lee et al., 2017). Based on this, Bae’s group fused the “magic bullets” MEL (GIGAVLKVLTTGLPALISWIKRKRQQQ) and natural antibacterial peptide dKLA(d [KLAKLAKKLAKLAK]) with a GGGGS linker to form MEL-dKLA peptide. They confirmed that MEL-dKLA minimized interaction with CD86^+^ M1 while killing CD206^+^ M2 (Lee et al., 2019).

However, the strong adverse hemolytic effect of MEL limited its application. To reduce toxicity, Soyoung Kim, another member of Bae’s group, synthesized and selected the best fragment termd VLTTGLPALISWIKRKRQQ. This fragment had dramatically decreased hemolytic effects but best specific M2 binding properties. Then the fragment fused with pro-apoptotic peptide d (KLAKLAK)2 (dKLA) by a GGGGS linker was modified with PEGylation at the amino terminus to form a more stable PEG-melittin-dKLA8-26. This modified fragment showed a potent therapeutic effect against cancers by depleting CD206+ M2 cells in Triple-Negative Breast Cancer. (Kim et al., 2022). The works of Bae’s group indicated the potential of MEL fragment in TAM targeting treatment.

**M2pep (M2 macrophage binding peptide)** is a targeted tool with high efficiency and specificity. The peptide sequence was identified by Maryelise Cieslewicz et al., in 2013 and has been shown to exhibit highly selective binding and internalization in M2 macrophages, making it a specialized M2 targeting structure (Cieslewicz et al., 2013).

In the existing applications, the M2pep usually functions together with α-peptide (a scavenger receptor B1 type (SR-B1) targeting peptide). For example, Qian et al. developed M2 dual-targeted nanoparticles (M2NPs) using this peptide, whose structure and function were controlled by the α-peptide connected to M2pep. Besides, they also loaded anti-colony stimulating factor-1 receptor (anti-CSF-1R) small interfering RNA (siRNA) onto M2NPs. Experiments have confirmed the effectiveness of the clearance receptor SR-B1 for M2 targeting and demonstrated that the synergy of the two targeting units (α-peptide and M2pep) in the fusion peptide (α-M2pep) made its affinity for M2 higher than that of other leukocytes (Yang et al., 2021). Based on the targeting functions of M2pep and α-peptide and the reversal of TAM type by baicalin and CpG-ODN, Han et al. invented a new type of nanoparticle that used poly (lactic-co-glycolic acid) (PLGA) to load baicalin and tumor-associated antigen Hgp100. Meanwhile, CpG-ODN was adsorbed on the surface of polydopamine (pD), using M2pep and αpep on the surface for targeting (Han et al., 2021). In addition, Parayath et al. established an HA-PEG carrier based on an HA-PEI carrier and surface-modified it with M2pep to increase targeting accuracy (Parayath et al., 2021).

**Specific hydrolyzed artificial peptides** can become a worthy restrictive peptide. Legumain asparaginyl endopeptidase is an overexpressed protein on M2. Jin et al. used a new hairpin-structured peptide that could be cleaved by legumain as a carrier to deliver simvastatin and paclitaxel in combination to dual-target the tumor microenvironment and regulate TME and TAM. This hairpin-structured peptide could be specifically cut by the legumain asparaginyl endopeptidase (Wang et al., 2018b), thus ensuring stability when the drug was off-target. The team used simvastatin as a payload, which has been shown to repolarize TAMs through cholesterol-related LXR/ABCA1 regulation, promoting the conversion from M2 to M1 phenotype (Jin et al., 2019).

### 2.3 Human proteins and peptides

In addition to actively targeting by modifying the carrier surface with exogenous peptide segments, using some human proteins and peptides as carriers or signaling molecules can also achieve targeting and ensure safety.

Human serum albumin (HSA) nanoparticles can target albumin-binding proteins (e.g., SPARC, secreted protein acidic and rich in cysteine), which have been shown to be overexpressed on M2 macrophages (Piris, 2021). As the intrinsic component in the human circulation system, HSA possesses the properties of stability, high biocompatibility and less cytotoxicity (Lu et al., 2022). Using basic targeted modification ideas, Feng et al. used palmitic acid-modified albumin nanoparticles as a delivery carrier for pirarubicin (THP), targeting both SPARC and scavenger receptor-A (SR-A). Experimental results showed that it had high selectivity and lethality for M2, and human albumin-based vectors did not cause liver or kidney damage (Feng et al., 2022). Moreover, the use of HSA can make some harmful or imprecise treatments relatively harmless. For example, one study proposed the use of HSA-modified nanogold particles (5b) to synthesize HSA-5b composite NPs to target and inhibit gastric cancer tumor-associated microenvironment cells (Zhang J. et al., 2021). Similarly, the excellent biocompatibility makes HSA promising adjuvants for photodynamic therapy. Lu et al. have developed photoactivated reactive oxygen species (ROS) responsive nanoplatforms, using anti-CD47 antibodies for active targeting and human serum albumin to construct the carrier. It has shown excellent stability and good photodynamic therapy efficacy (Lu et al., 2022). Except for photodynamic therapy, sonodynamic therapy can also be optimized using HAS. Ji et al. designed a mitochondria-targeted and ultrasound-responsive nanoparticle to co-deliver oxygen (O2) and nitric oxide (NO). The principle is to encapsulate perfluorodecalin (FDC) and sonosensitizer (IR780) with a human serum albumin-based NO donor (HSA-NO). The results showed that the drug could accumulate in mitochondria, relieve hypoxia, and actively repolarize M2 to M1 (Ji et al., 2021).

In addition, this human body aborigines display efficacy in passing through biological barriers. For example, Zhao et al. developed an albumin-based delivery system and used transferrin receptor-binding peptide T2 and mannose to co-modify nanoparticles, enabling them to pass through the blood-brain barrier and enhance targeting accuracy (Zhao et al., 2018).

**Antibody-conjugated nanocarriers** might be a possible targeting approach. On the one hand, the antibody can be conjugated with nanoparticles. For example, Sofie et al. developed an antibody-conjugated nanoparticle targeting the sialoadhesin receptor, mainly achieved by connecting polyethylene glycol (PEG) and polylactic acid-hydroxyethyl acid (PLGA) with anti-sialoadhesin monoclonal antibodies (Van Hees et al., 2022). On the other hand, the antibody may work as a marker on the surface of a membranous drug delivery platform. For instance, one study used a new monoclonal antibody that could bind CCL2 and CCL5 with dual specificity. The antibody was conjugated with a lipid nanoparticle platform (MC3 LNPs) encoding BisCCL2/5i mRNA, which could significantly induce polarization of TAMs towards an antitumor M1 phenotype and reduce immune suppression in the TME (Wang Y. et al., 2021).

Besides, another study using exosomes and monoclonal antibodies is very convincing. Nie et al. used M1-derived azo-modified exosomes and diphenylcyclooctane-modified CD47 and SIRP α antibodies (aCD47 and aSIRP α) connected by pH-sensitive linkers. While having targeting ability, the exosomes also had the ability to repolarize M2 (Nie et al., 2020). The above examples show that antibodies also have the characteristics of numerous targets and strong plasticity, and can target multiple targets and carry multiple drugs. However, there are still problems of physiological stability and immunogenicity in the use of antibodies, which makes it have disadvantages of a short half-life and ineffective drug use. As a “magic bullet”, it needs to be further studied (Wathoni et al., 2022).

## 3 TAM-assisting drug delivery system

Many synthetic nanomaterial therapies have been introduced above. However, despite great advancements in nanotherapeutics, there is still a long way to go in regard to clinical application. Biological stability, poor targeting, and rapid clearance from the body limit the effect of nanomedicine (Oroojalian et al., 2021; Khatoon et al., 2022). Recently, biomimetic approaches utilizing immune cells have emerged as promising strategy to solve these limitations. The fundamental properties of natural tumor-related immune cells, particularly the macrophages, allow the biomimetic platforms to cross the immune barriers, prolong their blood circulation time, avoid biodistribution in normal tissues, and reducetoxicity (Wu Y. et al., 2022; Lopes et al., 2022). Owing to its natural carrying capability for proteins and molecules, cells have drawn considerable attention for biomedical applications for designation of drug delivery systems (Li Z. et al., 2021). According to the characteristics, the aggregation of TAMs promotes the recruitment and accumulation of nanoparticles in the tumor sites. In addition, the leakage of TAMs into tumor tissues helps deepen the permeability of nanomedicines (Cheng Y et al., 2021). However, there are some obstacles in the application of natural cells due to their inefficient *in vivo* delivery and lack of therapeutic payloads. Therefore, chemical engineering provides a cost-effective, easy-to-implement and precision medicine-available strategy for the cell-based drug delivery systems (Wang et al., 2023). At present, there are almost three bioengineering applications based on the macrophage, including programmed live macrophages, macrophage-derived extracellular vesicles, and macrophage cell membrane-coated nanoparticles.

### 3.1 Programmed live macrophages

Owing to their intrinsic phagocytosis capacity, the live macrophage can be loaded with a variety of nanodrugs. Thus, the programmed macrophages can deliver nanomedicines to tumor sites while being recruited by the tumor. According to its loading place, this strategy can be mainly divided into two methods, *in vitro* loading and *in vivo* loading.

#### 3.1.1 *In vitro* loading

**FIGURE 3:**
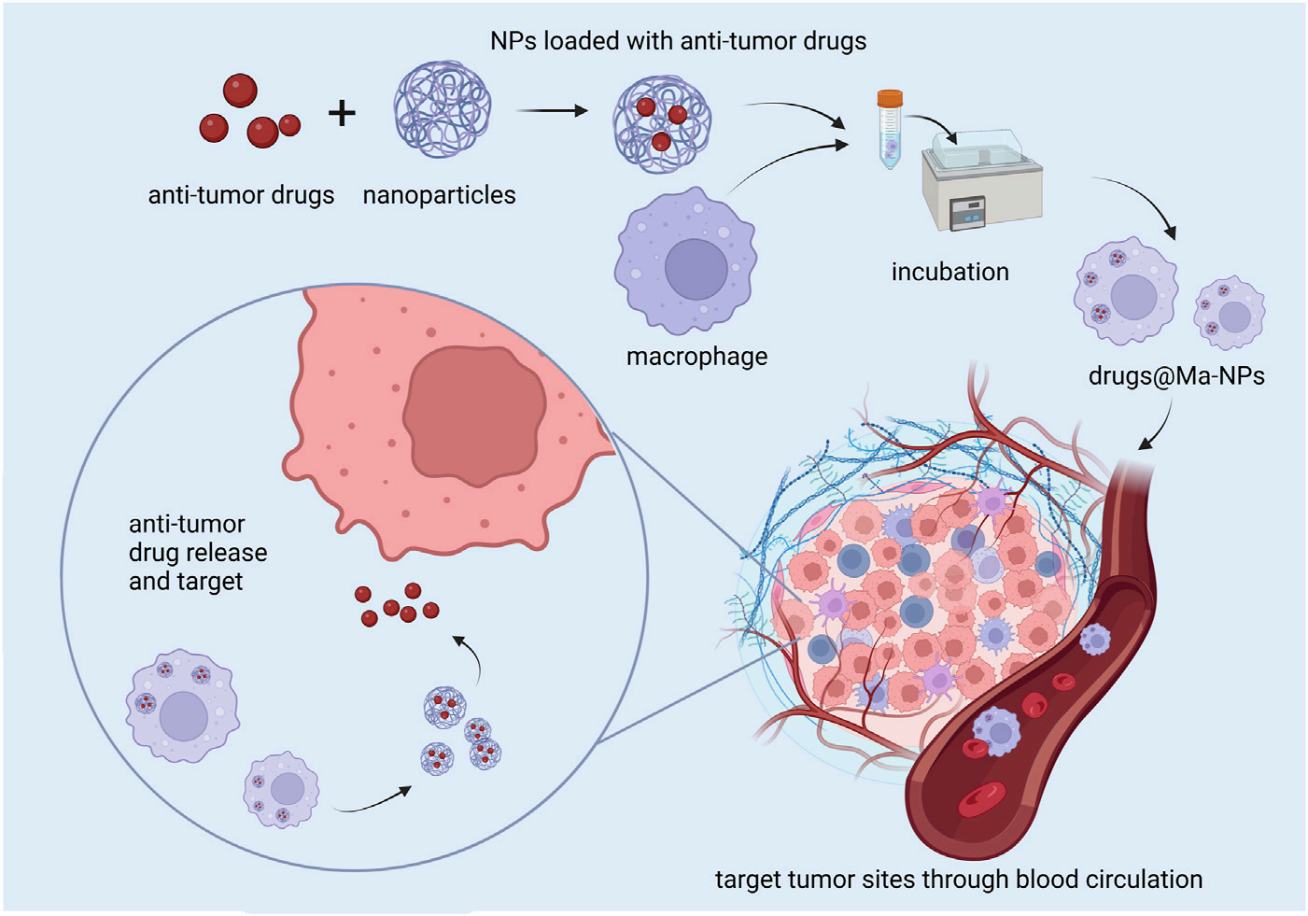
Basic route of TAMs assisting in the delivery of nanomedicines on *in vitro* loading.

Contemporary research about *in vitro* loading developed programmed live macrophages mainly by incubating macrophages with nanomedicines. After being injected into the tumor model, the macrophages can be recruited to the tumor tissues and release antitumor drugs (Figure 3) (Lopes et al., 2022).

For example, chemotherapy is indispensable in tumor therapies. However, the blood-brain barrier (BBB) severely restricts the access of most chemotherapy drugs to brain tumor sites. Therefore, we mainly take strategies aimed at brain tumors as an example to show the advance of programmed live macrophage platform. Compared to other types of macrophages, M1 macrophages with stronger phagocytic capability can load more drug-loaded nanoparticles. Pang et al. prepared M1 macrophage-loaded nanoparticles (M1-NPs) by incubating M1 macrophages with poly (lactide-co-glycolide) (PLGA) nanoparticles. The M1-NPs showed higher brain tumor distribution and greater system stability than free nanoparticles (Pang et al., 2018). In another study, M2 macrophages were chosen for glioblastoma (GBM) drug delivery. In this delivery system, polyglycerol (ND-PG) coated nanodiamonds (4–5 nm in diameter) were attached to cyclic tripeptides composed of L-arginine, glycine, and L-aspartic acid (RGD), a targeting moiety to integrin receptor αvβ3to form ND-PG-RGD. Loaded with doxorubicin (DOX), ND-PG-RGD turned into the ultimate product Nano-DOX. Finally, Nano-DOX-loaded M2 (Nano-DOX-M2) was prepared by incubating M2 with 2.5 μg/mL Nano-DOX for 24 h in a culture medium. In this study, it was demonstrated that Nano-DOX-M2 could successfully unload Nano-DOX into glioblastoma cells, resulting in the emission of damage-associated molecular patterns (DAMPs). In return, nano-DOX-damaged glioblastoma cells (GCs) exhibited an enhanced ability to recruit nano-DOX-loaded TAMs and reprogram them toward an anti-GC M1-like phenotype (Li T. F. et al., 2017).

Moreover, nanoparticles artificially reprogramming can also be applied to live macrophage. Chuxin et al. reported an artificial reprogrammed macrophage (HION@Macs) as a live cell-therapeutic for cancer suppression and immune recovery. In detail, they first developed hyaluronic acid-decorated superparamagnetic iron oxide nanoparticles (HIONs) by coating superparamagnetic iron oxide nanoparticles (IONs) with hyaluronic acid (HA). Then the nanoparticles were internalized into macrophages to form HION@Macs. It has been demonstrated that the high intracellular iron ions in macrophages could activate NF-kB and thus initiate a TNF-α associated proinflammatory response, effectively releasing inflammatory factors. In general, this study presents us how to reprogram natural macrophages with man-made materials and achieve bioactive cell therapeutics (Li et al., 2019). In addition, they also present us opportunities to get artificially arm live cells with additional functionalities as desired, such as the magnet-guided targeting, which may be further armed in other engineered cells.

Tracing the distribution and bio-behavior in the organism is vital for reducing macrophage therapy’s side effects (Sapach et al., 2022). As for the tracing, Yan et al. reported a new type of silver nanocluster (AgNC), which helped to track macrophages and stabilize their state. The subcellular localization examination showed that AgNCs escaped lysosomal degradation after endocytosis and presented stable fluorescence in macrophages, which contributed to macrophage-based drug delivery. In addition, AgNCs could also induce the M1-like polarization of macrophages through the Toll-like receptor 4 signaling pathway, enhancing the targeting capability. Therefore, AgNC-laden macrophages efficiently inhibited the growth of metastatic tumors (Yan et al., 2022).

Although the tumor-homing effect makes macrophage a good carrier for tumor targeting. However, when loaded in macrophages, nano-carriers are internalized and sequestered within the phagosomes, which may degrade the drugs and retard their release (Doshi et al., 2011). What’s more, it has been proved that some nanodrugs also show cytotoxicity for their carrier cells (Matic et al., 2020). Therefore, considering the toxicity and potential degradation of antitumor drugs, most of them cannot be directly loaded into macrophages.

Shields et al. developed a class of soft discoidal particles called “backpack” which can regulate macrophage phenotype of *in vivo*. Polyvinyl alcohol (PVA) was chosen as the interior layer between two poly (lactic-co-glycolic) acid (PLGA) layers to enable and stabilize incorporated IFN-γ. Then a cell-adhesive layer made by hyaluronic acid modified with aldehyde (HA-Ald) and poly (allylamine) hydrochloride (PAH) was added to help binding on macrophages. These particles could escape from phagocytosis and continuously release cytokines to regulate macrophages polarization, which could be maintained even in the strongly immunosuppressive environment. Therefore, the programmed live macrophages obtained excellent stability, keeping their function complete before releasing drugs in the tumor sites (Shields et al., 2020). In addition, a study reported a bio-orthogonal receptor approach to assemble polymeric prodrugs on genetically engineered Macrophages (GEMs) as a new type of cell backpacking platform. The receptor bound a fluorescein ligand that directed cell loading with ligand-tagged polymeric prodrugs, termed “drug gamers,” The ligand facilitated stable binding of drug gamer to engineered macrophages and the drugamers showed sustaining drug release and anti-proliferative activity against a glioblastoma cell line (Lopez et al., 2022). Besides, Cao et al. developed a macrophage-based delivery system (LD-MDS). LD-MDS was developed by conjugating the plasma membrane of live macrophage with a legumain-specific propeptide of melittin (legM) and cytotoxic soravtansine (DM4) prodrug. The platform showed great superior targeting efficiency for lung metastatic lesions. After infiltrating the tumor sites, the LD-MDS was activated by legumain protease and converted into DM4-loaded exosome-like nanovesicles (DENs) It was demonstrated that it displayed efficient internalization by metastatic 4T1 cancer cells and resulted in apoptosis and inhibition of lung metastasis (Cao et al., 2018). In addition, quantum dots and 5-(amino acetamido) fluorescein-labeled poly-amidoamine dendrimer G4.5, covered by amine-derivatized polyethylene glycol, were attached to the surface of macrophages through a transient Schiff base linkage (Holden et al., 2010). Azide-dibenzocyclooctyne can also be a good linker for connecting nanoparticles and macrophages (Hou et al., 2022).

#### 3.1.2 *In vivo* loading

Some nanomaterials are designed to target TAMs, which in turn to show their anti-tumor effects by tumor homing through TAMs. Thus, *in vivo* drug loading to macrophage is a considerable improvement strategy. Taken advantage of the specificity and targeting ability of rHDL on macrophages, Perez-Medina et al. developed an rHDL-based Zr-labelled TAM imaging agent.

With the recruitment of TAMs, the loaded nanomaterials were transferred to tumor tissues. Histological analysis after intravenous injection showed the co-localization of radioactivity with TAM-rich areas in tumor, which was sufficient to demonstrate the feasibility of this strategy in tumor imaging (Perez-Medina et al., 2015). PEGylated gold nanoparticles can also be used for fluorescence imaging of tumors. A related study found that after TAMs cross endothelial cells (N-TECs), they transported the nanoparticles from their exocytosis point to the tumor mesenchyme. Notably, this nano-system could theoretically also be loaded with some specific therapeutic molecules to achieve the anti-tumor effective (Lin et al., 2022). Another strategy relying on TME-sensitive cluster nanoparticles (SCNs) was developed by Shen et al. They developed ^BLZ945^SCNs/Pt which will undergo supersensitive structure collapse in the perivascular regions of tumor tissues. After structure collapse, platinum (Pt)-prodrug conjugated small particles and CSF-1R inhibitor BLZ945 were rapidly released. Then, BLZ945 preferentially absorbed by TAMs caused TAM dissipation. Meanwhile, small particles of Pt-precursor drug coupling penetrated deeper tumor tissues and released active Pt drug intracellularly to kill more tumor cells (Shen et al., 2017). The *in vivo* loading avoids the potential immune response caused by the heterogeneity of TAMs. However, the concrete mechanisms of *in vivo* targeting and loading remain to be explored.

### 3.2 Macrophage cell membrane-coated nanoparticles

At present, research in this field concentrates on designing biomimetic drug delivery platforms by coating nanoparticles with macrophage cell membranes. Combining the advantages of macrophage coating and nanodrugs, these platforms can obtain enhanced biocompatibility, namely, escaping from immune recognition to prolong their blood circulation time, and improved therapeutic efficacy owing to the natural cell functions (Wang et al., 2018a; Jiang et al., 2019).

Mostly, macrophage membrane-decorated liposomes were fabricated by just extruding the mixture solution of drug loading liposomes and purified macrophage membrane. The macrophage membrane coating emtansine liposomes developed by CaoHaiqiang et al. were reported to promoted the specific target ability to metastatic foci in the lung and suppressed lung metastasis of breast cancer (Cao et al., 2016). CaoXi et al. used paclitaxel (PTX) as model drug to develop drug loading albumin nanoparticles (ANPs) coated with macrophage plasma membranes (RANPs). In animal trials, this platform displayed enhanced cytotoxicity and apoptosis rates compared to bare albumin nanoparticles in the murine melanoma cell line B16F10 (Cao et al., 2020).

The kind of drug delivery system can also be combined with functional nanoparticles apart from antitumor drugs. For example, the macrophage membrane delivery system can load photosensitizer (PS), realizing TAM-membrane (TAMM)-based photodynamic immunotherapy. For the construction of TAM-like upconversion nano-PSs (NPR@TAMM), they first made NaYF4:Yb, Er@NaYF4 conjugated with Rose Bengal, producing a rare-earth-upconversion-nanoparticle (UCNP)-based PS (NPR). Then, the TAM membrane, derived from purified primary TAMs sorted by anti-F4/80 and CD206 beads, was coated onto NPR (NPR@TAMM), forming a platform consisting of a nanorod core ∼80 nm in length and an outer lipid bilayer shell ∼8 nm thick. This kind of photodynamic therapy (PDT)-immunotherapy platform utilized PSs to generate reactive oxygen species under light irradiation, thus killing tumor cells. Furthermore, the PDT-induced death of tumor cells released tumor-specific antigens, activating an antitumor immune response (Chen C. et al., 2021). Additionally, other research concentrated on the controlled release process of nanodrugs based on membrane-coating biotechnology. Li et al. designed a kind of TAM-loading nanodrugs (cskc-PPiP/PTX@Ma), which achieved a controlled release profile in response to TME stimuli. The macrophage membrane-coating was removed in response to the interstitial pH of TME. Guided by the insulin-like growth factor 1 receptor-targeting peptide, the released nanoparticles could be absorbed by tumor cells. As a reaction to the intracellular pH, the encapsulated drugs could be finally released from the nanoparticles, thus modulating tumor cell metabolism. In this way, the designed macrophage-membrane-coated nanomedicines exhibited penetration efficiency, better targeting capability, and enhancement of therapeutic effect, owing to the macrophage membrane coating and step-by-step drug release (Zhang et al., 2018).

Apart from single cell-derived membranes, hybridizing TAMs and other cell membranes to form composite membrane coating is also a promising strategy. It is demonstrated that the composite membrane carries properties of both source cells, thus achieving better targeting or delivering capability (Dehaini et al., 2017).

Malignant glioma is known for its chemotherapeutic tumor resistance and inaccessibility because of the blood-brain barrier (BBB). Yin et al. developed a biomimetic drug delivery platform called NMm-PLGA/RAPA by covering a rapamycin (RAPA)-loaded poly (lactic-co-glycolic acid) (PLGA) nanoparticle with neutrophil and macrophage membranes (NMm). Owing to the NMm coating, this platform could transmigrate across BBB and result in effectively and specifically targeting therapy to malignant glioma (Yin et al., 2022). In addition, to deal with triple-negative breast cancer (TNBC), which is known for its current lack of effective therapeutic targets, Zhang and his colleagues developed a macrophage/platelet hybrid membrane (LPHM) based biomimetic nanoplatforms with excellent TNBC-targeting ability. In their study, they first loaded large pores of dendritic large pore mesoporous silicon nanoparticles (DLMSNs) with a near-infrared (NIR) fluorescent dye IR780 and a chemotherapeutic drug doxorubicin (DOX) to develop DLMSN@DOX/IR780 (DDI) nanoparticles (NPs). Then the NPs incubated with LPHM in advance were sonicated to obtain LPHM@DDI NPs. In the trials, the obtained LPHM@DDI NPs had an excellent TNBC-targeting ability and showed cytotoxicity and apoptosis-inducing activity *in vitro* and *in vivo* PTT/PDT performances (Zhang T. et al., 2021). Besides, a recent study showed the possibility of macrophage-tumor hybrid membrane-coated nanoparticle use in drug delivery. First, the cell membrane of 4T1 breast tumor cells and RAW264.7 macrophage cells were purified and then fused by sonication. For the synthesis of nanoparticles, they added Dox to the solution of PLGA and incubating with stirring. Then Dox-PLGA were mixed and sonicated with a macrophage-tumor hybrid membrane to prepare membrane-camouflaged NPs. Compared with the research above, this platform showed homogenous tumor targeting abilities *in vitro* via α4 integrin-VCAM-1 interaction and markedly enhanced multitarget capability in a lung metastasis model *in vivo* owing to the participation of tumor cell membranes (Gong et al., 2020). In addition, the coating of the hybridized cell membranes prolongs the circulation time of delivery platforms and presents an antimetastatic effect.

### 3.3 Macrophage-derived extracellular vesicles (EVs)

Macrophage-derived extracellular vesicles exhibit intrinsic cancer or inflammation-targeting properties owing to their particular membrane proteins which inherit from macrophages (Li S. et al., 2020). Besides, naturally, macrophage-derived extracellular vesicles, especially exosomes, specialize in long-distance intercellular communications. Therefore, as endogenous vesicles, exosomes diminish clearance by the mononuclear phagocyte system (MPS), as well as avoid toxicity and immunogenicity, ensuring a long blood circulation time (Xiong et al., 2019; Li S. et al., 2020). However, native exosomes usually induce insufficient effects *in vivo* (Nie et al., 2020). Therefore, the artificial macrophage-derived extracellular vesicles may be a potential drug-delivering strategy owing to their unique tumor-targeting ability.

As we mentioned above, there are obstacles to the efficient targeted delivery of anticancer agents to breast tumors. Macrophage-derived EVs possess an extraordinary ability to interact with and accumulate in cancer cells. Herein, we mainly focus on the application of macrophage-derived extracellular vesicles in this field.

Based on previous study, Haney et al. reported a manufacture and characterization of macrophage-derived EVs based drug formulation approach with different loading procedures optimized with pH, temperature, and sonication conditions. They developed a EVs based platform loaded with chemotherapeutic agents, paclitaxel (PTX), and doxorubicin (Dox). Which showed efficient target ability and antitumor effect toward orthotopic mouse T11 tumors in immune-competent BALB/C mice, and human MDA-MB-231 tumors in athymic nu/nu mice (Haney et al., 2020).

In order to enhance the targeting ability, on the one hand, peptide modification is an idea. Li et al. designed a macrophage-derived exosomes-coated poly (lactic-co-glycolic acid) nanoplatform. Especially, a peptide, which targeted the mesenchymal-epithelial transition factor (c-Met) overexpressed by TNBC cells, was anchored to the surface of the exosome. It displayed tumor-targeting efficacy and induced intense tumor apoptosis (Li S. et al., 2020). On the other hand, antibody modified responsive nano-bioconjugates exosome could be a strategy as well. To prepare the responsive exosomes, azide-modified exosomes derived from M1 macrophages were conjugated with dibenzocyclooctynes modified with aCD47 and aSIRPα antibodies. Through the specific recognition between antibodies and antigens, the exosomes could actively target the tumor sites. The aSIRPα and aCD47 can be released from the exosomes due to acidic TME which will result in abolishing of “do not eat me” signaling and enhanced phagocytosis effect of macrophages, respectivly (Nie et al., 2020).

In addition, another study also used the aCD47 as a modification. The researchers developed an injectable hydrogel scaffold loaded with engineered exosome mimetics. These M1 macrophage-derived exosome mimetics were conjugated with vesicular stomatitis virus glycoprotein (VSV-G) and aCD47 (V-M1EM-aCD47). The hydrogel recruited intrinsic macrophages and released V-M1EM-aCD47 which was capable of reprogramming M2 to M1-aCD47 macrophages. The tumor-homing of M1-aCD47 macrophages could make it possible for the delivery of aCD47 that blocked the “do not eat me” signal, thereby promoting phagocytosis of macrophages to cancer cells (Wu et al., 2023).

Except for loading chemotherapy drugs, EVs also have been applicated in other types of therapies. For instance, sonodynamic therapy (SDT) has shown its efficiency in glioblastoma (GBM) treatment. However, the barrier function of the blood−brain barrier (BBB) limits access to GBM to a great extent. In a recent study, a biodegradable nanoplatform (CSI) was developed by encapsulating catalase (CAT) into silica nanoparticles (CAT@SiO2), and then loaded with the sonosensitizer indocyanine green (ICG). What’s more, CSI was coated with AS1411 aptamer-modified macrophage exosomes to form CSI@Ex-A. It has been demonstrated that CSI@Ex-A displayed efficiency in BBB penetration and tumor targeting. After internalization, the intracellular glutathione (GSH) promoted biodegradation of the nanoplatform and the released CAT catalyzed hydrogen peroxide (H2O2) to produce O2, improving tumor hypoxia (Wu T. et al., 2022). The programmed exosomes can also be used in the treatment of lung tumors. The upconversion nanoparticles (UC) modified with mesoporous silica (SUC) were used to load suberoylanilide hydroxamic acid (SAHA). SAHA was an inhibitor of histone deacetylase (HDAC), whose overexpression, usually regarded as epigenetic dysregulation, was associated with malignant tumors. And the product was further camouflaged with M1 macrophage-derived exosome membranes (EMS). EMS displayed special spatiotemporal-resolved properties and increased the drug accumulation in the tumor sites. When taken up by lung cancer cells, the platform could effectively inhibit HDAC activity, presenting an excellent antitumor effect (Li H. et al., 2021). Several exosome-based platforms have been worked out as drug carriers (Hou et al., 2023; Lee and Lee, 2023; Li et al., 2023; Li and Luan, 2023; Yu et al., 2023).

At present, the exosome isolation techniques still need improving with production and functional property loss (Rayamajhi et al., 2019). To solve this problem, Rayamajhi et al. developed a hybrid exosome (HE) by hybridizing macrophage-derived exosomes and synthetic liposomes. Biochemistry techniques showed that HE was a vesicle of 177 ± 21 nm with EV marker proteins CD81, CD63, and CD9. The water-soluble doxorubicin-loaded HE showed pH-sensitive drug release properties in acidic TME and enhanced cytotoxicity (Rayamajhi et al., 2019). Another research provided a different idea. Here, Jang et al. produced the bioinspired exosome-mimetic nanovesicles by using a serial extrusion through filters with diminishing pore sizes (10, 5, and 1 μm) to split the macrophages. It showed a 100-fold higher production yield than natural exosomes. It was demonstrated that the purified nanovesicles contained exosomal marker proteins like CD63 and Tsg101. Meanwhile, the macrophage-derived nanovesicles shared a similar size, morphology, targeting and delivery ability with exosomes (Jang et al., 2013).

## 4 Conclusion and perspectives

Macrophages, derived from monocytes, play a prominent role in the development of diseases, especially in tumor progression. Specifically, in recent years, TAMs have been widely researched in nano-related immunotherapy focused on tumor treatment. In contrast to traditional nanomaterials, present TAM-based nanomedicines have higher specificity, fewer side effects, and a better prognosis. With research development, an increasing number of target sites and pathways have been discovered. To date, there are three main mechanisms of TAM-based nanomedicines.

1) Inhibiting the recruitment of macrophages by limiting the effect of vascular endothelial growth factor (VEGF) or blocking TAM-recruiting pathways. As a result, it hinders the formation of TAMs and then inhibits tumor angiogenesis, tumor immunoregulation, etc.
2) Suppressing survival of TAMs by targeting the delivery of cytotoxic drugs.
3) Transforming TAM phenotypes from M2 with immunosuppression to M1 phenotypes shows an antitumor effect.

Herein, we also discuss some approaches to increase the targeting capacity of nanomedicines, including carbohydrate ligands and short peptides. Conjugated with targeting drug delivery platforms, they can promote the recognition and adhesion between nanomedicines and target cells. Moreover, in terms of the properties of macrophage membranes and the mobility of TAMs, they are reprogrammed as an efficient drug delivery platform, which means the capability of crossing immune barriers, prolonged blood circulation time, and lower toxicity. Additionally, the recruitment of TAMs promotes the accumulation of nanomedicines in tumor sites, leading to a high level and better effects of drugs. The deep soakage of TAMs helps enhance the permeability of nanomedicines by transferring them to areas such as hypoxic areas and tumor stem cells, which is difficult for other delivery platforms to approach.

As we mentioned above, TAMs participate in the tumor treatment mainly through two strategies. On the one hand, the nanomedicines can be designed to directly targeting the TAMs, leading to further influence on tumor growth. In this way, the drugs reshape the TME and hinder tumor progression. However, the biodistribution in normal tissues and inefficiency in infiltrating into tumor sites still remain to be solved. Compare with the former one, the TAM-based delivery platforms prolong the circulating time and promote the permeation into deep of the tumor tissues. But there may exist potential immune risks. In summary, nanomaterials for tumor therapy related to tumor-associated macrophages as target spots or delivery platforms are promising and efficient methods for tumor treatment. However, more research is needed to support further clinical applications and address the limitations and disadvantages.

In the tables, we list latest TAM-targeted nanomedicines laboratorial projects and clinical trials. These drugs are designed to interfere with the biological activity of tumor-associated macrophages and are expected to play an important role in tumor therapy. We have listed their names, targets, indications, effects and related clinical trial progresses (Table 2). To some degree, this information helps researchers obtain a more comprehensive picture of nanodrugs. However, most of them are at an early stage of pre-clinical or clinical trials, and there are still a series of limitations for researchers.

**TABLE 2:** TAM-targeted nanomedicines in clinical trials.

Target	Compounds	Tumor type	Clinical phase	NCT number	Ref
CSF-1R	Pexidartinib/PLX3397	Tensynovial cell giant tumor	Approved by FDA in 2019 for the treatment of TGCT	NCT02371369	Tap (2020)
	ARRY-382	non-small cell lung cancer (NSCLC)	Phase 1b/2	NCT02880371	Harb et al. (2017)
	DCC-3014	tenosynovial giant cell tumor	Phase 1/2	—	Taylor et al. (2019)
	PLX7486 TsOH	solid tumors	Phase 1	NCT01804530	—
TLRs	Resiquimod	melanoma	Phase 1/2	NCT01810016	Sabado et al. (2012)
PI3K	IPI-549	Advanced solid tumors	Phase 1 b	NCT02637531	Sullivan et al. (2018)
CCR2	Carlumab	Metastatic Castrate-Resistant Prostate Cancer	Phase 2	NCT00992186	—
CD40	ChiLob7/4	pancreatic cancer and head and neck cancer	Phase 2	NCT01561911	Li and Wang (2020)
	ABBV-428	Pancreatic cancer; lung cancer; ovarian cancer	Phase 1	NCT02955251	
CD47	Hu5F9-G4-Rituximab	Non-Hodgkin’s Lymphoma	Phase1/2	NCT02953509	
C/EBPα	MTL-CEBPA	Hepatic cancer	Phase1	NCT02716012	
			Phase1	NCT04105335	
PD-1	Pembrolizumab	Breast Cancer	Phase3	NCT02819518	
	BA3011/CAB-AXL-ADC	Solid Tumour	Phase1/2	NCT03425279	
		Non-Small Cell Lung Cancer			
		Castration-Resistant Prostate Cancer			
		Pancreatic Cancer			
AXL	CCT301	Renal Cell Carcinoma	Phase1/2	NCT03393936	
	AVB-S6-500	Ovarian Cancer	Phase1	NCT03401528	
		Primary Peritoneal Cancer	Phase1	NCT03639246	

Note: Data were obtained from https://clinicaltrials.gov/.

For example, although we have mentioned strategies to increase the targeting capacity of nanomedicines above, more attention should be given to this field since the surface markers vary in macrophages and different cells, including macrophages and other immune cells, share several kinds of receptors. It somehow discourages nanomedicines from recognizing target cells. In addition, on the one hand, the lack of a strong specific delivery mechanism may lead to the uptake of nanomaterials by cells in other parts of the body. On the other hand, the drug release speed is difficult to control, which may affect the therapeutic effect and safety of the drug. In this regard, researchers have tried to design a drug release mechanism related to temperature and pH and have achieved some accomplishments. However, whether it can be further applied is still waiting for more extensive research (Liaw et al., 2021). Ultimately, safety testing of the relevant material and drug combination is still in the laboratory stage, and there is still a certain distance from clinical trials.

For the magic bullets that can actively target TAMs, although they have come a long way since being used as an alternative strategy for drug-targeted delivery, there is still some room for growth. For example, the mannose receptor has been widely used as a target for TAMs. However, it is also present in dendritic cells and some endothelial cells as a highly efficient endocytic receptor, with potential off-target and side effects. It is therefore possible to add the restriction of targeting TME to existing drugs or to find new TAM-exclusive targets to improve the effectiveness of the magic bullet. Among the studies using peptides as carriers, M2pep, which is discovered 10 years ago, is still being used as an important tool. At present, most of the studies are based on the modification of this peptide, indicating that there is still much room for exploration.

Regarding macrophage membrane-based delivery platforms, the heterogeneity of TAMs, determined by the origins of macrophages, is also in the way. The immune response between macrophage membrane suppliers and receptors remains unknown due to its variety. Moreover, the modification of the membrane and the transformation of physicochemical properties during membrane separation, purification, and coating may lead to an unconceived effect. In addition, the detailed route and distribution of nanomedicines remain to be explored. Despite the nano-based imaging noted above, more effective visual image technology is expected to tackle this problem.

In summary, we reviewed some important and latest applications of TAM-based antitumor nanomedicines, displaying the significance and great potential of TAMs in the treatment of tumor. Research mentioned in this review may help improve existent designs or even provide new research orientation. Besides, considering the promising application of delivery systems to revolutionize the oncotherapy, present limitations and disadvantages await urgently to be solved, which encourages more exploration and reformation in this field.
